# Antibiotic resistance in *K. pneumoniae*: perspectives, challenges, health risks, therapeutic approaches

**DOI:** 10.3389/fmicb.2026.1816741

**Published:** 2026-08-17

**Authors:** Tülay Kandemir, Shiva Eivazi Bagheri, Gamze Ayakdaş, Duygu Ağagündüz, Bence Raposa, Fatih Ozogul

**Affiliations:** 1 Biotechnology Research and Application Center, Cukurova University, Adana, Türkiye; 2 Faculty of Pharmacy, Çukurova University, Adana, Türkiye; 3 Department of Nutrition and Dietetics, Acibadem Mehmet Ali Aydinlar University, Istanbul, Türkiye; 4 Department of Nutrition and Dietetics, Gazi University, Ankara, Türkiye; 5 Institute of Basics of Health Sciences, Midwifery and Health Visiting, Faculty of Health Sciences, University of Pécs, Pécs, Hungary; 6 Department of Seafood Processing Technology, Faculty of Fisheries, Cukurova University, Adana, Türkiye

**Keywords:** epidemiology, *Klebsiella pneumoniae*, resistance mechanisms, surveillance, therapeutic approaches, virulence

## Abstract

*Klebsiella pneumoniae* is an opportunistic Gram-negative pathogen of increasing global concern due to its expanding virulence potential and rapid acquisition of antimicrobial resistance. Traditionally associated with hospital-acquired infections such as pneumonia, urinary tract infections, and bloodstream infections, *K. pneumoniae* has evolved into a highly diverse species comprising classical (cKP), hypervirulent (hvKP), multidrug-resistant (MDR), and emerging carbapenem-resistant hypervirulent (CR-hvKP) variants. Its pathogenicity is driven by a wide array of virulence factors, including capsular polysaccharides, siderophores, fimbriae, lipopolysaccharides, and multiple secretion systems, all of which facilitate immune evasion, tissue invasion, biofilm formation, and environmental survival. Parallel to this, *K. pneumoniae* has developed extensive resistance through intrinsic mechanisms, such as efflux pumps and porin alterations, as well as acquired determinants including ESBLs, carbapenemases (KPC, NDM, and OXA-48), 16S rRNA methyltransferases, and plasmid-mediated *mcr* genes conferring colistin resistance. These combined traits have led to widespread dissemination of MDR and XDR strains, limiting therapeutic options and contributing to significant morbidity, mortality, and healthcare burdens worldwide. Epidemiologically, resistance hotspots have emerged in Asia, Europe, and the Middle East, while hvKP continues to expand beyond its original endemic regions. Diagnostic challenges, therapeutic limitations, and economic pressures further complicate clinical management. This review synthesizes current knowledge on *K. pneumoniae* virulence mechanisms, resistance evolution, epidemiology, and clinical impact, and highlights emerging therapeutic strategies aimed at overcoming this escalating public health threat.

## 1 Introduction

*Klebsiella pneumoniae* is a rod-shaped, encapsulated, non-motile, Gram-negative bacterium belonging to the Enterobacteriaceae family. Characterized by a prominent polysaccharide capsule that confers a mucoid phenotype, this opportunistic pathogen accounts for more than 95% of all clinical infections attributed to the genus *Klebsiella* (Martin and Bachman, 2018; Ferreira et al., 2018; Shah et al., 2025). *K. pneumoniae* is implicated in a broad spectrum of human diseases, most notably lower respiratory tract and urinary tract infections, as well as life-threatening bloodstream infections. While historically considered a predominantly nosocomial threat affecting hospitalized or immunocompromised individuals, the global epidemiology of *K. pneumoniae* has undergone a critical shift, solidifying its status as a major public health crisis worldwide (Martin and Bachman, 2018).

The clinical management of *K. pneumoniae* has become profoundly complicated by the rapid evolution and dissemination of antimicrobial resistance (AMR). The pathogen employs a sophisticated arsenal of resistance mechanisms, including the production of extended-spectrum beta-lactamases (ESBLs) and carbapenemases, efflux pump upregulation, and genetic mutations altering porins and penicillin-binding proteins (PBPs). As a prominent member of the infamous “ESKAPE” group of pathogens, *K. pneumoniae* exhibits a remarkable capacity for horizontal gene transfer. Plasmids serve as the primary vehicles driving the co-dissemination of multidrug resistance (MDR) genes and key virulence factors such as siderophores, fimbriae, lipopolysaccharides (LPS), and capsules (Ferreira et al., 2018; Dong et al., 2022).

Based on distinct phenotypic and genetic traits, *K. pneumoniae* isolates are classified into three major clinical archetypes: classical (cKP), hypervirulent (hvKP), and multidrug-resistant (MDR) variants (Li L. et al., 2023). The cKP strains are most frequently encountered in healthcare settings, showing a strong propensity to acquire resistance plasmids carrying carbapenem resistance genes, with regional prevalence rates exceeding 10% in certain areas (Ferreira et al., 2018; Lei et al., 2024). Conversely, hvKP strains are characterized by unique virulence profiles that allow them to evade host immune defenses, causing severe community-acquired infections, such as pyogenic liver abscesses, meningitis, and endophthalmitis, in otherwise healthy individuals (Martin and Bachman, 2018).

The phenotypic hallmark of hvKP is elevated mucoviscosity during *in vitro* agar cultivation, clinically validated by a positive string test. Genetically, these strains are predominantly restricted to the K1 and K2 capsular serotypes and are strongly governed by specific regulatory loci, most notably the regulator of mucoid phenotype A (rmpA) and the mucoviscosity-associated gene A (magA). Furthermore, contemporary investigations have identified the production of aerobactin, a high-affinity siderophore that enhances iron-scavenging capabilities, as a primary virulence factor and a crucial diagnostic indicator distinguishing hvKP lineages (Guo et al., 2017). Mortality rates in hvKP infections have historically been reported to range from 3 to 32% (Ferreira et al., 2018).

The most alarming evolutionary development in recent years is the convergence of these phenotypes, giving rise to hybrid pathotypes that combine hypervirulence with carbapenem resistance (CR-hvKP) (Lei et al., 2024; Song et al., 2025). These hybrid strains typically emerge via three evolutionary scenarios: (1) carbapenem-resistant *K. pneumoniae* (CRKP) strains acquiring virulence plasmids; (2) classical strains (cKP) integrating composite plasmids that carry both resistance and virulence genes; or (3) hvKP strains (most frequently serotypes KL1 and KL2) gaining carbapenem resistance plasmids (Lei et al., 2024). These hybrid pathotypes severely restrict remaining therapeutic options, compromise the efficacy of last-line agents, and amplify their clinical impact (Lei et al., 2024).

Recent epidemiological data highlight the escalating global burden of these resistant strains. Current global modeling data and long-term projections indicate that *K. pneumoniae* has become one of the leading bacterial causes of mortality worldwide, associated with approximately 800,000 total deaths annually. Critically, roughly 80% of this total mortality load is directly linked to antimicrobial resistance, with regional MDR and carbapenem-resistant prevalence rates reaching up to 60% in high-burden areas (Martin and Bachman, 2018; Song et al., 2025). Consequently, the World Health Organization (WHO) continues to list carbapenem-resistant Enterobacteriaceae, including *K. pneumoniae*, as a critical priority threat, especially given that general AMR-associated mortality spans millions of deaths globally (Lei et al., 2024).

Beyond its biological virulence, *K. pneumoniae* thrives in clinical environments due to its robust ability to form dense biofilms on indwelling medical devices (e.g., ventilators and catheters), protecting the bacteria from both host immune clearance and systemic antibiotics. Efficient transmission within healthcare environments is further facilitated by contaminated instruments, surfaces, or the hands of healthcare staff, particularly in hospitals with high patient density or inadequate infection control. Patients in intensive care units (ICUs) are especially susceptible because of invasive procedures, multiple comorbidities, and extended hospitalization (Li et al., 2025b).

CRKP has been recognized as one of the leading bacterial causes of mortality linked to antimicrobial resistance, contributing to a massive global death toll annually, with resistance rates escalating rapidly over the past two decades (Qala Nou et al., 2025; Zeng et al., 2023). Reports of hvKP are also increasing globally, with the highest burden observed in East and Southeast Asia, including countries like China, Taiwan, South Korea, and Iran. For example, a multicentre study in China demonstrated that 22.8% of invasive *K. pneumoniae* infections were attributable to hvKP (Guo et al., 2017). Overall, hvKP represents a substantial proportion of circulating isolates, frequently exhibiting co-resistance to ESBLs and colistin, with associated pooled mortality rates around 28% (Qala Nou et al., 2025).

These hybrid CR-hvKP strains present serious clinical challenges, with mortality rates ranging between 30 and 60%, and morbidity reaching 60%−90%, especially in hospital settings (Jesudason, 2024). The spread of resistance via plasmids renders many standard therapies, including carbapenems, ineffective. While polymyxins are occasionally used, their nephrotoxicity limits their utility, and tigecycline shows poor efficacy in bloodstream infections due to suboptimal plasma concentrations. New beta-lactamase inhibitors like ceftazidime-avibactam are costly and not readily available in low-income countries, which increases the burden on healthcare systems and leads to prolonged ICU stays. Furthermore, access to advanced alternative options like monoclonal antibodies and bacteriophage therapy remains limited in many regions (Jesudason, 2024).

The economic ramifications are equally severe, with the estimated direct drug cost per patient infected with *K. pneumoniae* carbapenemase-producing strains reaching approximately $4,100, with about 60% of this cost incurred during the acute phase of illness (Santos and Secoli, 2019). This urgent threat is also addressed in the Updated Global Action Plan on Antimicrobial Resistance (2026–2036), which reinforces international efforts to curb AMR-attributable mortality through strict stewardship (Jesudason, 2024). Based on these data, this review aims to comprehensively examine antimicrobial resistance in *K. pneumoniae*, which has emerged as a major public health problem worldwide. In addition to detailing its mechanisms of resistance, diagnostic approaches, and epidemiology, this review uniquely highlights cutting-edge emerging treatment developments, including novel next-generation antibiotics, phage therapy, CRISPR-based strategies, and nanotechnology, alongside modern methods to prevent and control the spread of resistance and *K. pneumoniae* virulence factors.

## 2 *K. pneumoniae* virulence factors

### 2.1 Capsule

**Figure 1:**
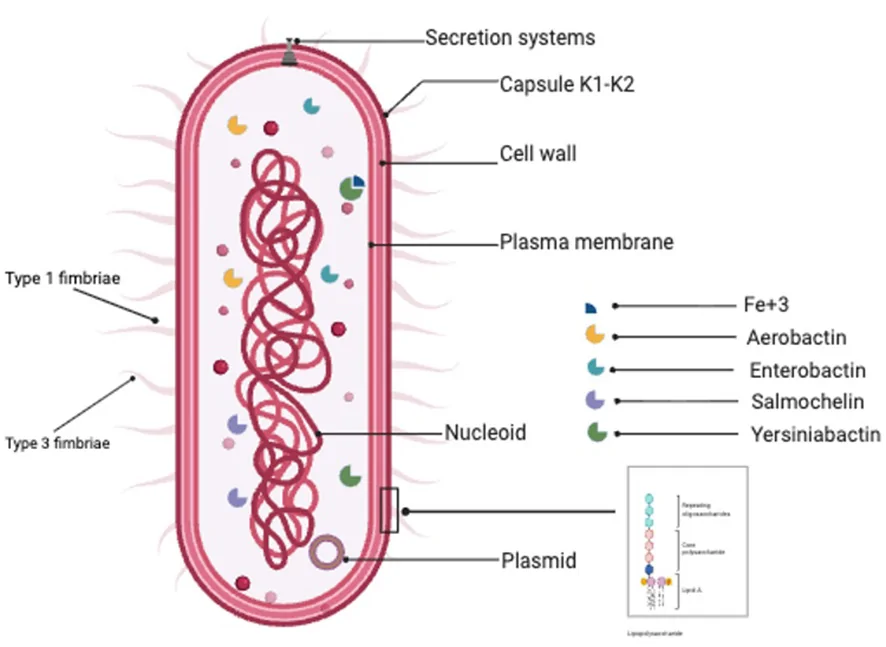
*K. pneumoniae* virulence factors. Created with BioRender.

The capsule constitutes the primary virulence factor responsible for the development of the hypermucoviscosity phenotype (Monteiro et al., 2024). It covers the cell surface. and is composed of a polysaccharide matrix. One of the functions of the capsule is to provide resistance to antibacterial peptides by blocking access to the bacterial outer membrane, thus protecting it from the bactericidal effect of antibacterial peptides (Fleeman et al., 2020). Additionally, the capsule protects bacterial cells from phagocytosis (Singh et al., 2025). Capsule polysaccharide, which has negatively charged phagocyte-like properties, repels phagocytes and protects against phagocytosis. It escapes host complement-mediated opsonophagocytosis by masking membrane-associated proteins and cell wall structures. The bacteria are protected from oxidative destruction by increasing their resistance (Huang et al., 2022). Despite the capsule being recognized as a major virulence factor and an attractive target for vaccine development in both Gram-positive and Gram-negative bacteria, no licensed vaccine currently exists to prevent *K. pneumoniae* infections. Nevertheless, the capsular structure of *K. pneumoniae* remains a promising candidate for vaccine design (Ravinder et al., 2020). The genetic determinants responsible for capsule biosynthesis are located within the chromosomal capsular polysaccharide synthesis (cps) locus, which contains more than twenty genes. Among these are *wzc, wzb, wza, wzi, wzx, wzy, wbaP, wcaJ, manC*, and *galF* (Han et al., 2024; Shu et al., 2019). Specifically, genes such as *wzc, galF, cpsACP, wzi, wza*, and *wzb*, which play critical roles in cps translocation, modification, and surface protein transport, are situated in the 5′ region of the *cps* gene cluster. The *gnd* and *ugd* genes are located in the 3′ region. The genes located between the 5′ and 3′ regions encode various glycotransferases that provide capsule diversity. The genes encoding capsule polysaccharide subunits are *wzx, wzy, wzc, wcaJ*, and *wbaP*. Glycosyltransferases play an essential role in catalyzing the assembly and polymerisation of polysaccharide subunits that constitute the capsule. Among the capsule-associated genes, *wzc, wzx*, and *wzy* are universally present in all capsular serotypes, although their sequences differ considerably across strains. The specific capsule type in which the *wcaJ* and *wbaP* genes are found depends on which sugar initiates the capsule synthesis process. The *wcaJ* and *wbaP* genes encode a glycosyltransferase enzyme that initiates capsule synthesis. *K. pneumoniae* capsule polysaccharides consist of sugar units, with the length of these sugar units varying between 3 and 6 sugars. These sugars include mannose, fucose, rhamnose, and galactofructose. The *manB* and *manC* genes code for the synthesis of mannose sugar (Singh et al., 2025; Pan et al., 2015). *K. pneumoniae* carries a 200-kb virulence plasmid linked to hypervirulence. This virulence plasmid encodes positive regulators of capsule biosynthesis and siderophores. To date, more than 130 distinct capsule types have been identified in *K. pneumoniae*, and the variation among these capsules plays a critical role in determining the bacterium's virulence. Capsule types are systematically classified as either K or KL. Notably, hvKP strains are frequently associated with particular capsule types, with K1 and K2 being the most commonly reported among highly virulent isolates (Dorman et al., 2018; Wu et al., 2023). Six capsule types, K57, K54, K20, K5, K2, and K1, are linked to hypervirulence and pyogenic liver abscesses. Additionally, *K. pneumoniae* isolates with these K types are responsible for approximately 70% of community-acquired pneumonia cases (Wu et al., 2023) (Table 1 and Figure 1). In *K. pneumoniae* infections, the high pathogenicity of hvKP strains is directly linked to the overproduction of the polysaccharide capsule; this mechanism is rigorously controlled by specific genetic loci (Dorman et al., 2018). The most prominent of these regulators are the *rmpA* gene and the *rmpA2* gene, which are usually found in the pathogen's large virulence plasmid (pLVPK). The rmpA gene is considered a critical determinant of pathogenicity (Dorman et al., 2018; Han et al., 2022; Lin et al., 2020; Cheng et al., 2010). At the molecular level, this gene strongly stimulates the cps gene cluster responsible for capsule synthesis during transcription, triggering the formation of a large polysaccharide armor around the bacterium. This allows the bacterium to evade the immune system. *rmpA* also functions as a global transcription regulator due to its LuxR-like receptor domains (Singh et al., 2025; Cheng et al., 2010; Xu et al., 2024). This genetic factor determines the physiology of the pathogen by comprehensively reprogramming cellular metabolism. In particular, it coordinates vital transitions between the stages of bacterial hypermucoviscosity and biofilm formation. This versatile genetic control mechanism allows *K. pneumoniae* to seamlessly adapt to extremely diverse and challenging environmental conditions, making this gene a potential and highly attractive target for future next-generation antimicrobial therapies (Singh et al., 2025; Dorman et al., 2018; Yao et al., 2025).

**Table 1:** Major virulence factors of *K. pneumoniae*, biological functions, and clinical outcomes.

Virulence factor	Functional role in pathogenicity	Biological function	Clinical outcomes	References
Capsular polysaccharide (CPS)	Immune evasion, resistance to phagocytosis, serum resistance	Prevents complement depositionInhibits phagocytosis by macrophages and neutrophilsEnhances survival during oxidative stressHypermucoviscosity in hvKP strains	Invasive infections (liver abscess, pneumonia, sepsis) Severe disease linked to K1/K2 capsules High mortality in hvKP infections.	Monteiro et al., 2024; Fleeman et al., 2020; Singh et al., 2025; Huang et al., 2022; Han et al., 2024; Shu et al., 2019; Dorman et al., 2018; Wu et al., 2023
Siderophores (Aerobactin, Enterobactin, Salmochelin, Yersiniabactin)	Iron acquisition and immune evasion	Scavenge iron from host proteinsAerobactin enhances virulence dramatically (93%−100% in hvKP)Salmochelin resists lipocalin-2 defenseYersiniabactin supports systemic spread	Increased severity of bloodstream infections Pyogenic liver abscess (especially aerobactin and salmochelin) Enhanced dissemination and organ invasion	Monteiro et al., 2024; Han et al., 2024, 2022; Lin et al., 2020; Li J. et al., 2018; Kumar et al., 2024; Davidov et al., 2025
Fimbriae (Type 1 and Type 3)	Adhesion, biofilm formation, colonization of tissues & devices	Type 1 The FimH facilitates lectinophagocytosisType 3: MrkA/MrkD promote biofilm formation on abiotic surfacesEssential for UTI invasion and colonization	Catheter-associated UTIs Device-associated pneumonia and bacteremia Chronic biofilm-driven infections with increased antibiotic tolerance	Monteiro et al., 2024; Han et al., 2024; Assoni et al., 2025
Lipopolysaccharide (LPS)	Complement evasion and inflammatory response modulation	Lipid A interacts with innate immunityO-antigen blocks C1q binding, allowing serum resistanceCore oligosaccharide highly immunogenic	Severe sepsis and septic shock Increased survival in bloodstream infections Drives strong inflammatory cytokine responses	(Han et al., 2024; Remya et al., 2019; Bulati et al., 2021; Miller et al., 2024)
Secretion systems (T1SS–T6SS)	Delivery of toxins, host manipulation, bacterial competition	T1SS: secretes enzymes/toxinsT2SS: secretes proteases, lipasesT3SS: injects effectors (rare in Kp)T4SS: transfers DNA → spreads resistance/virulenceT5SS: autotransporters aiding adhesionT6SS: injects toxic effectors; promotes interbacterial killing	Increased virulence and tissue destruction High colonization efficiency (T6SS in gut) Facilitates MDR dissemination via T4SS hvKP often T6SS-associated (Pld1, Tle1 effectors)	Singh et al., 2025; Hetta et al., 2025; Green and Mecsas, 2016; Korotkov et al., 2012; Maphosa et al., 2023; Shaliutina-Loginova et al., 2023; Naskar et al., 2021; Yang et al., 2023; Prince and Wong Fok Lung, 2023; Luo et al., 2024; Meuskens et al., 2019; Liu et al., 2017; Merciecca et al., 2022; Liao et al., 2022
Allantoin metabolism	Nutrient acquisition (carbon and nitrogen) in host tissues	More common in K1 hvKPEnhances survival under nutrient limitation	Strongly linked to liver abscess formation Higher invasiveness and metastatic infection potential	Monteiro et al., 2024; Chou et al., 2004; Kim et al., 2017
Peg-344	Marker of hypervirulence	May encode an inner membrane transporterAssociated with plasmid-borne virulence islandsNot required for systemic infection but linked to organ targeting	Correlates with hvKP infections Associated with invasive diseases (liver abscess, meningitis)	Hetta et al., 2025; Bulger et al., 2017

hvKP, hypervirulent *K. pneumoniae*; UTI, urinary tract infection; T1SS, Type 1 Secretion System; T2SS, Type 2 Secretion System; T3SS, Type 3 Secretion System; T4SS, Type 4 Secretion System; T5SS, Type 5 Secretion System; T6SS, Type 6 Secretion System; MDR, Multi-Drug Resistant.

### 2.2 Siderophores

Iron is an essential nutrient for both the host and *K. pneu*moniae, playing a crucial role in their metabolic functions. In the host, iron is primarily sequestered within proteins such as ferritin, transferrin, haptoglobin, and hemoglobin. Siderophores are low-molecular-weight proteins that, once synthesized intracellularly, are secreted into the extracellular environment where they bind iron and facilitate its uptake into the bacterial cell (Monteiro et al., 2024). During infection, the availability of iron to *K. pneumoniae* is limited, prompting the bacterium to develop specialized mechanisms to acquire this nutrient. Through a siderophore-mediated iron acquisition system, *K. pneumoniae* extracts iron from host iron-binding proteins, such as transferrin. hvKP are capable of producing four distinct siderophores: aerobactin, salmochelin, enterobactin, and yersiniabactin, which collectively enhance their virulence and survival within the host environment (Han et al., 2024, 2022). Aerobactin is an important virulence factor frequently (93%−100%) found in hvKP isolates and rarely in cKP isolates. Detection of aerobactin in an isolate indicates that the isolate is 96% hvKP (Lin et al., 2020). The biosynthesis of aerobactin in *K. pneumoniae* is mediated by the *iucABCD* gene cluster. The *iutA* gene encodes the outer membrane receptor for aerobactin and serves as a molecular marker indicating the presence of this siderophore in bacterial isolates. Other associated marker genes include *iucA, iucB, iucC*, and *iucD*. Aerobactin has been frequently detected in clinical infections caused by *K. pneumoniae*, particularly in isolates obtained from blood, liver abscesses, urinary tract infections, and other systemic infections, with a high prevalence reported in studies from Asian populations (Monteiro et al., 2024; Li J. et al., 2018).

Enterobactin constitutes the primary iron acquisition system in *K. pneumoniae*. The *entABCDEF* gene cluster governs the biosynthesis of enterobactin, while the *fepABCDG* cluster encodes the components necessary for enterobactin transport (Han et al., 2024). Production of enterobactin is commonly observed in both cKP and hvKP strains of *K. pneumoniae* (Kumar et al., 2024). Salmochelin is a glycosylated derivative of enterobactin that is particularly prevalent in hvKP strains, especially those implicated in pyogenic liver abscesses, where its expression rate approaches 90%. The glucosylated modified form prevents the binding of lipocalin-2 to salmochelin. Lipocalin-2, which blocks iron uptake by bacteria, is a glycoprotein that induces inflammation and neutralization (Han et al., 2024; Jaberi et al., 2021). Salmochelin is commonly detected in hvKP strains but is infrequently observed in cKP isolates. The *iroA* gene cluster encodes the biosynthesis of salmochelin and its corresponding receptor. Molecular markers used to identify this siderophore include the *iroA* and *iroN* genes. The hypervirulence traits and cellular preservation mechanisms dictated by the *iroA* locus depend on the synchronized functions of *iroB, iroC, iroD, iroE*, and iroN. While the surface-bound receptor *IroN* operates as the primary gateway for importing iron-bound salmochelins, the remaining four genetic units drive the metabolic manufacturing and processing steps. Concretely, IroB serves as the transforming enzyme that links glucose molecules onto the core enterobactin frame. This alteration creates a protective spatial barrier that prevents host defense proteins, specifically siderocalin, from trapping the pathogen's iron collectors in systemic circulation. Once generated, *IroC* functions as an internal barrier pump, directing these uncomplexed chelators outward into the extracellular environment to intercept environmental ferric ions. Following the successful recovery of iron via *IroN*, the internalized molecule undergoes structural cleavage to liberate the bound nutrient. This breakdown is executed by two separate lactone-splitting proteins, *IroE* and *IroD*, which exhibit structural similarity to the classic Fes enzyme. To maximize processing efficiency through spatial segregation, *IroE* initiates early degradation within the periplasmic compartment, whereas *IroD* completes the final reduction inside the cytoplasm, safely delivering Fe^3+^ to the microbial metabolic machinery (Lin et al., 2005). In addition to their role in iron acquisition, salmochelins can inhibit the growth of competing bacterial species (Monteiro et al., 2024; Davidov et al., 2025). Yersiniabactin represents the second most prevalent siderophore identified in *K. pneumoniae*. This siderophore is found in about half of the cKPs and almost all of the hvKPs. The gene cluster that controls its expression is *ybtAEPQSTUX*, also recognized as *ybt*, along with the *irp* genes (*irp1* and *irp2*). The genes encoding its synthesis are *irp* genes; the gene encoding receptor uptake is *ybtQ*; and the genes facilitating its transport are *ybt* and *fyu*. In many studies, *ybtA, ybtS, irp1, irp2*, and *fyuA* genes have been used as markers to identify this siderophore (Monteiro et al., 2024; Han et al., 2024) (Table 1 and Figure 1). These mobile genetic elements responsible for yersiniabactin production (e.g., ICEKp10) not only facilitate iron acquisition through horizontal gene transfer in the pathogenic evolution of *K. pneumoniae* isolates but also integrate into the cellular genome, leading to the acquisition of dangerous genotoxins such as colibactin. This genotoxin, generally encoded via the same genetic island (ICE) as yersiniabactin, is considered one of the key triggers of severe abscesses in tissues, particularly those caused by hypervirulent strains (e.g., the CG23 subspecies) (Hetta et al., 2025). Colibactin causes significant tissue damage by directly damaging DNA and leading to cell death in infected host cells. Furthermore, clinical epidemiological data have revealed a statistically significant increase in the incidence of colon cancer in patients following cases of pyogenic liver abscesses caused by this genotoxin-producing pathogen. This striking situation has given rise to the hypothesis that colibactin may function not only as an invasive infectious agent but also as a potential carcinogenic agent triggering mutations in host cells (Hetta et al., 2025; Russo et al., 2023).

### 2.3 Fimbriae

Considering the gene clusters identified in the *K. pneumoniae* genome, it is thought to possess ten different types of fimbriae. The gene clusters *kpg, kpf, kpe, kpd, kpc, kpb*, and *kpa* have been identified so far. However, information about these clusters is still insufficient. *K. pneumoniae* primarily expresses type 1 and type 3 fimbriae as its main adhesins (Monteiro et al., 2024). The primary fimbriae found in hvKP and cKP are types 1 and 3. These membrane-bound fimbriae consist of both structural and adhesive subunits. The subunits of type 1 fimbriae in *K. pneumoniae* are encoded by genes regulated by the fim cluster, whereas the *mrk* gene clusters control type 3 fimbriae subunits. These fimbrial structures, located on the bacterial surface, play a pivotal role in biofilm formation, enabling the bacteria to adhere to both biotic and abiotic surfaces (Monteiro et al., 2024; Han et al., 2024). Type 1 fimbriae are filamentous appendages composed of protein subunits, notably including FimH and FimA, which contribute to their adhesive properties. The protein that facilitates lectinophagocytosis, a phenomenon performed by neutrophils and macrophages, is FimH. It also attaches to immune cells, such as mast cells, to stimulate the host's immune response. With this binding, neutrophils that contribute to bacterial clearance are attracted to the infection site. *K. pneumoniae* strains with type 1 fimbriae can both invade bladder cells and form biofilms during urinary tract infections in the bladder, due to these fimbriae (Monteiro et al., 2024; Han et al., 2024; Assoni et al., 2025). Type 3 fimbriae in *K. pneumoniae* are helical structures composed primarily of MrkD and MrkA subunits. MrkD serves as an adhesin, mediating interactions with components of the extracellular matrix. At the same time, MrkA forms the polymeric shaft of the fimbria and also contributes to adhesion to abiotic surfaces, thereby promoting biofilm formation. Additionally, type 3 fimbriae may stimulate neutrophils to increase the production of reactive oxygen species, leading to oxidative stress within the host (Assoni et al., 2025). Strains expressing type 3 fimbriae can efficiently colonize medical devices, such as catheters and ventilators, through biofilm-mediated adherence. The biofilm not only facilitates attachment but also provides *K. pneumoniae* with protection against host immune defenses. Additionally, through biofilm as a physical barrier, the resistance of bacteria to antibiotics increases by 10 to 1000 times, allowing them to continue to survive and grow (Monteiro et al., 2024; Han et al., 2024) (Table 1 and Figure 1).

### 2.4 Allantoin metabolism

Allantoin metabolism represents a biochemical pathway utilized by *K. pneumoniae* to obtain both carbon and nitrogen from allantoin. Allantoin itself is a byproduct of purine catabolism, serving as a substrate that can be further processed to support bacterial growth and survival. This metabolic pathway involves several enzymes, expressed by two transcriptional regulators (A1IR and A1IS) and three operons. The *allo* gene cluster regulates this metabolism. Unlike commensal strains, this gene cluster is more commonly found in strains linked to pyogenic liver abscess. The prevalence is 100%, especially in strains of the K1 serotype (Monteiro et al., 2024; Dai and Hu, 2022). The gene responsible for activating this system is the *allS* gene, which serves as a marker for the allantoin mechanism (Monteiro et al., 2024; Dai and Hu, 2022; Remya et al., 2019). Studies have demonstrated that deletion of the *allS* gene in *K. pneumoniae* reduces liver abscess formation and attenuates bacterial virulence in murine models (Chou et al., 2004). Globally, the *allS* gene is detected in approximately 13.6% of *K. pneumoniae* isolates (Kim et al., 2017). Its prevalence in liver abscess-associated strains varies considerably among studies, ranging from 40 to 100%. In contrast, *K. pneumoniae* strains responsible for urinary tract infections exhibit a much lower frequency of *allS*, with reported rates ranging from 0.58 to 40% (Monteiro et al., 2024).

### 2.5 Lipopolysaccharide

LPS is an essential component of the outer membrane in both cKP and hvKP strains, playing a central role in bacterial virulence. The *uge* gene, which encodes UDP-galacturonate 4-epimerase, regulates LPS biosynthesis and has been linked to the pathogen's ability to cause urinary tract infections, pneumonia, and sepsis (Remya et al., 2019). LPS is structurally composed of three primary regions: the O-antigen, the core oligosaccharide, and lipid A. The O-antigen, encoded by the *wb* gene cluster, functions as a protective shield by preventing C1q binding and facilitating the removal of C3b from the bacterial surface, thereby helping the bacterium evade complement-mediated killing. The core oligosaccharide, located nearer the bacterial membrane, is highly immunogenic and serves as a candidate for vaccine development (Han et al., 2024; Bulati et al., 2021; Miller et al., 2024). Lipid A, encoded by the *lpx* cluster, offers resistance to cationic antimicrobial peptides (Han et al., 2024) (Table 1 and Figure 1).

### 2.6 Bacterial secretion systems

Secretion systems are critical for *K. pneumoniae* survival and pathogenicity. These protein secretion systems play a crucial role in mediating host tissue damage, evading the immune response, facilitating horizontal gene transfer, and promoting bacterial adhesion to various surfaces (Singh et al., 2025; Green and Mecsas, 2016) (Table 1 and Figure 1).

#### 2.6.1 Type 1 secretion system

The Type 1 Secretion System (T1SS) comprises three fundamental components: An outer membrane protein (OMP), a membrane fusion protein, and an inner membrane ATP-binding cassette (ABC) transporter. This system enables the direct, single-step translocation of toxins, enzymes, antibiotics, and other effector molecules into the extracellular environment, thereby enhancing bacterial adaptability and virulence. T1SS is widely observed among *K. pneumoniae* strains (Singh et al., 2025; Korotkov et al., 2012; Maphosa et al., 2023).

#### 2.6.2 Type 2 secretion system

*K. pneumoniae* employs the Type 2 Secretion System (T2SS) to enhance environmental adaptability (Green and Mecsas, 2016). This system mediates the translocation of folded proteins, with quaternary structures, from the periplasmic space to the extracellular milieu. T2SS represents an evolutionarily conserved machinery among Gram-negative bacteria and is believed to have evolved from an ancestral structure involved in the biogenesis of type IV pili. Additionally, it shares evolutionary links with the formation of pili, flagella, and other surface appendages in archaea (Singh et al., 2025; Shaliutina-Loginova et al., 2023). It forms channels to transport Tat-dependent or Sec-dependent proteins to the outside of the cell. It facilitates the transfer of various proteins, including glycan proteins, phosphatases, lipases, and proteases, that contribute to virulence. The T2SS is composed of four main components: the outer membrane complex (secretin), the inner membrane platform, the pseudopilus, and the secretory ATPase (Singh et al., 2025; Naskar et al., 2021). The pseudopilus is a fibrous structure located within the periplasmic space, whereas the outer membrane complex consists of a multimeric protein assembly. The inner membrane platform is instrumental in the T2SS mechanism, orchestrating interactions with the outer membrane complex, the pseudopilus, and the secretory ATPase, while overseeing the transport of substrates across the cell envelope (Singh et al., 2025; Shaliutina-Loginova et al., 2023).

#### 2.6.3 Type 3 secretion system

A key characteristic of the Type 3 Secretion System (T3SS) is its ability to translocate bacterial effector proteins directly into host cells. These effector proteins modulate host cellular processes, allowing the bacterium to manipulate host cell functions to its advantage. This contributes to the virulence and colonization of the bacteria. The T3SS is called an “injectisome” or “molecular needle” due to its anatomical structure (shape) and the way it contacts the host cell to transport the protein. With its complex supramolecular structure, the T3SS traverses both bacterial and host cell membranes to reach its host target in a single step.

Furthermore, the T3SS is structurally and evolutionarily related to the bacterial flagellar apparatus. Since it is a system generally acquired by horizontal gene transfer, similar components are encountered even in evolutionarily distant species. Although the T3SS is a significant contributor to virulence in numerous pathogens, it has not been identified in *K. pneumoniae* strains in several investigations. Further research in this area is warranted (Singh et al., 2025; Yang et al., 2023; Prince and Wong Fok Lung, 2023).

#### 2.6.4 Type 4 secretion system

Type 4 secretion system (T4SS), evolutionarily connected to the bacterial DNA conjugation system, enables the transport of intercellular single protein molecules, protein-protein, and DNA-protein complex molecules from one bacterium to another host bacterium or the cytoplasm of eukaryotic cells. One of the main functions of this system, which transfers both DNA and proteins, is the transfer of conjugated DNA. Therefore, since the material must be delivered directly to the host cytoplasm, the integration of the donor cell DNA into the host cell DNA is relevant. Additionally, the transport of effector proteins is facilitated through macromolecular complexes, such as DNA-protein complexes (Singh et al., 2025; Green and Mecsas, 2016). T4SS is composed of twelve components, including VirD4 and VirB1–VirB11. VirB3 and VirB6 through VirB10 form the backbone of the system, while VirB2, together with VirB5 proteins, forms the extracellular pilus. The T4SS plays a critical role in *K. pneumoniae* by mediating the transfer of genetic material, thereby contributing to increased virulence and antimicrobial resistance. This secretion system is particularly prevalent in MDR strains (Singh et al., 2025; Green and Mecsas, 2016; Yang et al., 2023). A recent study demonstrated that horizontal gene transfer facilitated by T4SS, involving plasmids carrying resistance determinants, significantly enhanced the survival and adaptability of *K. pneumoniae* (Yang et al., 2023).

#### 2.6.5 Type 5 secretion system

The Type 5 secretion system (T5SS) consists of only one polypeptide chain. In Gram-negative bacteria, it spans only one layer of the outer membrane. The T5SS does not have an ATP source in the periplasm or an energy source, such as proton or ion gradients, in the outer membrane. It is a fully autonomous system; therefore, it is designated as an autotransporter (Luo et al., 2024). The characteristics of the T5SS include a right-handed and β-helical structure. It secretes large protein molecules. The protein to be extracted entirely from the cell forms a channel-like structure inside the outer membrane of Gram-negative bacteria, such that either a part of a protein or a separate protein is transported. While the primary structures and synthesis patterns of proteins using the T5SS have common similarities, their functional properties vary. It is a system generally used for the secretion of substances, including serine proteases, cytotoxins, lipases, invasins, and adhesins, which affect the ability of bacteria to grow in specific environments, form aggregates and biofilms, and their virulence. In addition, the T5SS, which plays a role in a phenomenon called contact-dependent growth inhibition, inhibits the growth of another competing bacterium by bringing it into contact with its own toxins (Singh et al., 2025; Luo et al., 2024; Meuskens et al., 2019). Despite being much smaller than other bacterial secretion systems, the T5SS is crucial for adhesion, pathogenicity, intercellular communication, and nutrient absorption. However, more research is required to clarify the function and underlying mechanisms of T5SS in *K. pneumoniae* because of the paucity of available data on this species (Singh et al., 2025; Meuskens et al., 2019).

#### 2.6.6 Type 6 secretion system

The Type 6 Secretion System (T6SS) is a key virulence determinant in *K. pneumoniae* strains. Functioning analogously to a nanosyringe, T6SS enables the bacterium to inject effector proteins directly into eukaryotic host cells or competing prokaryotic cells, resulting in the disruption of target cell structures. The presence of T6SS effectors has been associated with the hypervirulent phenotype of *K. pneumoniae* (Hetta et al., 2025). The first T6SS effector identified in *K. pneumoniae* was Pld1, which belongs to the phospholipase D family. Studies have shown that mutant strains with a deletion of the *pld1* gene are avirulent compared to the wild type (Han et al., 2024). Overexpression of Tle1, a T6SS effector in *K. pneumoniae*, has been shown to inhibit the growth of competing bacteria, such as *Escherichia coli* (Liu et al., 2017). T6SS also plays a critical role in the successful colonization of *K. pneumoniae* within the gastrointestinal tract (Merciecca et al., 2022). Strains harboring T6SS exhibit enhanced drug resistance, increased biofilm-forming capacity, and elevated virulence potential (Liao et al., 2022).

### 2.7 Peg-344

One of the most clinically critical genetic biomarkers associated with the development of the hypervirulence phenotype in *K. pneumoniae* is the peg-344 gene (Hetta et al., 2025; Bulger et al., 2017). Typically found in large virulence plasmids along with other important hypervirulence markers such as rmpA, magA, and aerobactin, this gene plays a crucial role in the molecular pathogenesis of this pathogen (Bulger et al., 2017). While its precise biochemical function remains unclear, *in silico* analyses suggest that peg-344 encodes a specific intramembrane metabolite transporter (Hetta et al., 2025; Bulger et al., 2017), a permease belonging to the drug/metabolite transporter (DMT) superfamily. This transporter is thought to directly facilitate the cellular uptake of essential growth factors that the bacterium needs in its challenging host environment (Bulger et al., 2017). This gene mediates highly specific, organ-directed pathogenicity (Hetta et al., 2025). *In vivo* mouse models have shown that deletion of peg-344 does not compromise the bacterium's ability to cause systemic or subcutaneous infections but significantly reduces its virulence in pulmonary infections, indicating that peg-344 is absolutely essential for pulmonary pathogenesis (Hetta et al., 2025; Bulger et al., 2017). Accurate differentiation between cKP and high-morbidity hvKP strains is vital in clinical practice. Therefore, the highly conserved and genetically stable structure of the peg-344 gene makes it an excellent target for rapid molecular diagnostic tests, including polymerase chain reaction and loop-mediated isothermal amplification (LAMP; Table 1) (Liao et al., 2020).

## 3 Mechanisms of antibiotic resistance in *K. pneumoniae*

### 3.1 Intrinsic vs. acquired resistance

Bacteria utilize various mechanisms to survive infection. Some of these mechanisms are intrinsic, while others are acquired. Resistance is defined as the continued ability of bacteria to multiply in the presence of an antimicrobial (Braun et al., 2024). At the same time, resistance represents a natural adaptive process that applies selective pressure on bacteria. Consequently, it cannot be wholly eradicated; however, it can be mitigated. Despite modifications in treatment protocols, AMR continues to evolve dynamically. The WHO considers this situation a reversion to the pre-antibiotic era (Hasan et al., 2021). Intrinsic resistance refers to resistance originating from chromosomally located gene clusters that are independent of the antimicrobials to which bacteria have previously been exposed. Furthermore, these gene clusters are not acquired via horizontal gene transfer. In *K. pneumoniae*, intrinsic mechanisms of AMR include efflux pumps and reduced cell wall permeability. Water-filled protein channels in the outer membrane, known as porins, facilitate the transport of small hydrophilic molecules, including antibiotics, nutrients, and iron. The bacterial capsule contributes to resistance by preventing antimicrobial peptides, including polymyxin-like agents, from reaching the cell surface. In this context, the capsule plays a significant role in antimicrobial resistance. Additionally, the ability of *K. pneumoniae* to form biofilms enables it to adhere to both biotic and abiotic surfaces, thereby protecting against the effects of various antimicrobials, including β-lactams, fluoroquinolones, and aminoglycosides (Braun et al., 2024; Huy, 2024). Chromosomally encoded SHV-1 penicillinase confers intrinsic resistance to ampicillin, ticarcillin, and amoxicillin, while both SHV-1 and TEM-1 enzymes mediate inherent resistance to penicillins such as ampicillin (Moya and Maicas, 2020; Kakoullis et al., 2021). Beyond these intrinsic mechanisms, *K. pneumoniae* also acquires additional resistance through mutations or horizontal gene transfer. Mutations at antimicrobial target sites prevent drug binding, and resistance genes acquired via mobile genetic elements, including plasmids and transposons, enable the bacterium to hydrolyse various antimicrobial agents, thereby broadening its resistance spectrum (Karami-Zarandi et al., 2023; Li Y. et al., 2024).

### 3.2 Key resistance mechanisms

#### 3.2.1 Efflux pumps

Bacterial efflux pump systems are crucial mechanisms that contribute to both AMR and virulence. Due to their broad-spectrum activity, mutations that disrupt efflux pump function can restore bacterial susceptibility to various antimicrobial agents (Braun et al., 2024). Efflux pumps are classified into five prominent protein families: Resistance-Nodulation-Division (RND), Major Facilitator Superfamily (MFS), ATP-Binding Cassette (ABC), Small Multidrug Resistance (SMR), and Multidrug and Toxic Compound Extrusion (MATE) (Karami-Zarandi et al., 2023). In *K. pneumoniae*, the most prominent efflux systems are AcrAB-TolC and OqxAB, both of which belong to the RND family. Chromosomal gene clusters encode these pumps and contribute to intrinsic resistance against antibiotics and antiseptics. They also play a role in intestinal colonization and the dissemination of pathogenic determinants. Structurally, RND efflux pumps consist of three components that span the inner membrane, periplasm, and outer membrane. In the AcrAB-TolC system, AcrB functions as the inner membrane transporter, TolC forms the outer membrane channel, and AcrA acts as the periplasmic accessory protein (Ashwath, 2021; Al-Dahmoshi et al., 2022; Wang et al., 2017).

The TolC and AcrB proteins, located in the outer and inner membranes, respectively, interact to form the functional AcrAB-TolC efflux system, with AcrA serving as a periplasmic bridge connecting the two components. AcrB captures substrates from the cytoplasm or the inner membrane phospholipid bilayer and translocates them through TolC across the outer membrane (Braun et al., 2024; Al-Dahmoshi et al., 2022). The *AcrR* and *RamR* genes act as transcriptional repressors of this system; deletion mutations in these genes result in overexpression of AcrAB-TolC. Such overexpression confers resistance to a broad spectrum of antimicrobials, including β-lactams, macrolides, tetracyclines, fluoroquinolones, chloramphenicol, and disinfectants in *K. pneumoniae* (Karami-Zarandi et al., 2023; Li Y. et al., 2024). The OqxAB efflux system is encoded on both chromosomal and plasmid DNA. Overexpression of OqxAB reduces susceptibility by more than fourfold, resulting in resistance to quinolones, tigecycline, and phenicols (Karami-Zarandi et al., 2023; Li Y. et al., 2024; Li et al., 2019). Other RND superfamily members found in *K. pneumoniae* include *EefAB, KexD, pmexAB-oprY*, and *tmexCDI-toprJ* (Karami-Zarandi et al., 2023; Ashwath, 2021). The genes encoding the EefAB efflux pump are located on the chromosome and contribute to the development of resistance to tetracycline, oxacillin, norfloxacin, erythromycin, and novobiocin (Karami-Zarandi et al., 2023).

The genes encoding the KexD efflux pump are chromosomally located, and overexpression of KexD confers resistance to tetracycline, macrolides, aztreonam, and tobramycin (Maurya et al., 2019). Colistin resistance is regulated by the CrrAB two-component system, where mutations in the *CrrB* gene induce KexD expression, resulting in colistin resistance (Sun et al., 2020).

Overexpression of the pmexAB-oprY efflux pump has been linked to tigecycline resistance. The genes encoding pmexAB-oprY are carried on IncFIA and IncFIB plasmids. When these plasmids also harbor the *mcr* (*mobile colistin resistance*) gene, responsible for colistin resistance, the bacterium can develop simultaneous resistance to both tigecycline and colistin (Karami-Zarandi et al., 2023; Sun et al., 2020).

Additionally, the TmexCDI-toprJ efflux system, encoded on plasmids and identified in animal isolates of *K. pneumoniae*, confers resistance to tigecycline, quinolones, cephalosporins, aminoglycosides, and chloramphenicol. Co-location with the *mcr-8* gene on IncFIA/IncFII plasmids further mediates colistin resistance (Qu et al., 2023).

The ABC efflux pumps utilize the energy derived from ATP hydrolysis to expel multiple drugs from the bacterial cell. In *K. pneumoniae* isolates resistant to tigecycline, the *IS5* insertion element enhances the expression of the *kpgABC* gene cluster, and overexpression of this cluster reduces tigecycline susceptibility by approximately fourfold. YbtPQ is another ABC transporter encoded within the highly mobile *ICEKp1* (integrative conjugative element) in *K. pneumoniae*. Deletion of *ICEKp1*, a 62-kb chromosomal island present in roughly half of clinical *K. pneumoniae* isolates, increases resistance to antibiotics such as gentamicin, doxycycline, ofloxacin, rifampicin, trimethoprim, and imipenem (Karami-Zarandi et al., 2023; Farzand et al., 2021, 2019).

The MFS represents the largest group of secondary membrane transporters, primarily involved in the uptake of sugars. Specific MFS proteins also function in drug efflux, contributing to antimicrobial resistance. While sugar transport generally involves a single-component pump, MFS efflux systems consist of three components: an inner membrane protein, a periplasmic protein, and an OMP. Notable MFS efflux pumps in *K. pneumoniae* include SmvA, SmvR, and KpnGH. SmvA mediates biocide resistance, and its overexpression or loss of the regulatory *smvR* gene results in biocide resistance (Karami-Zarandi et al., 2023; Pasqua et al., 2019). Overexpression of the multidrug MFS pump KpnGH, composed of KpnG and KpnH subunits, confers resistance to quinolones, carbapenems, aminoglycosides, macrolides, piperacillin, and polymyxin B. Additionally, KpnGH enhances bacterial survival under conditions of severe osmotic and oxidative stress (Pasqua et al., 2021).

**Figure 2:**
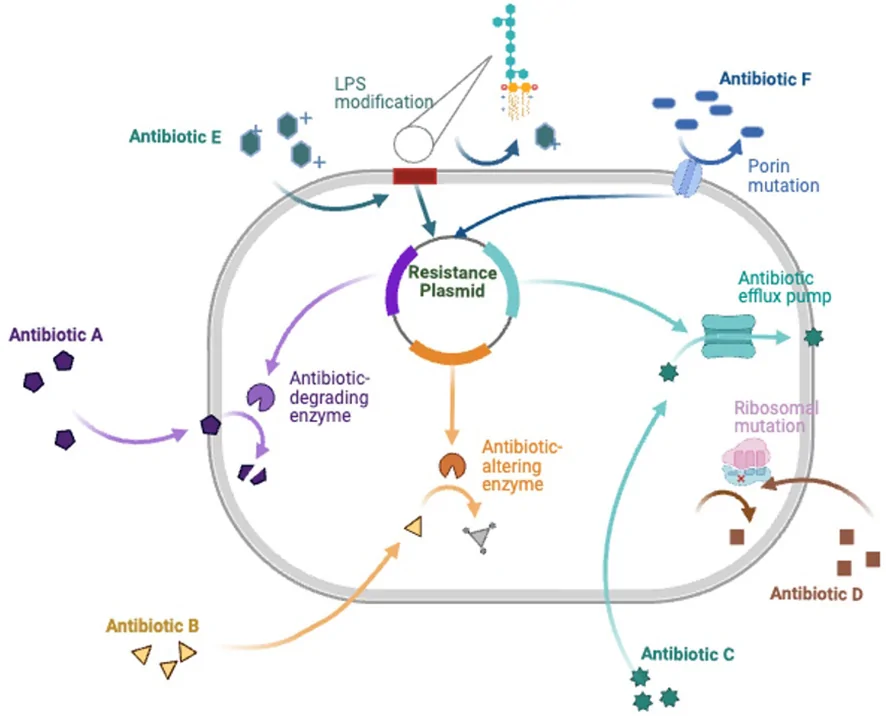
Key resistance mechanisms in *K. pneumoniae*.

The smallest efflux proteins belong to the SMR multidrug efflux pump family. The KpnEF efflux system, a member of the SMR family, is encoded chromosomally in *K. pneumoniae* (Karami-Zarandi et al., 2023). KpnEF facilitates the transport of polysaccharides to the outer membrane, contributing to biofilm formation and the development of mucosal structures (Ding Y. et al., 2023). Additionally, it plays a role in capsule biosynthesis. Mutations in KpnEF have been associated with decreased susceptibility to cefepime, tetracycline, rifampin, erythromycin, streptomycin, colistin, and ceftriaxone (Davin-Regli et al., 2021). The integration and modification of diverse substrates are critical for bacterial survival, and the MATE efflux pump participates in these processes. It also contributes to resistance against quinolones, aminoglycosides, and various dyes (Al-Dahmoshi et al., 2022). Genes encoding the KdeA, KetM, and MdtK efflux pumps, which are members of the *K. pneumoniae* MATE efflux pump family, are located on the chromosome. Expression of KdeA confers resistance to norfloxacin, acriflavine, ethidium bromide, and chloramphenicol; meanwhile, the activity of KetM and MdtK confers resistance to cefotaxime, acriflavine, norfloxacin, and ciprofloxacin (Karami-Zarandi et al., 2023) (Figure 2).

#### 3.2.2 Target site modifications

Mutations at the target sites of antimicrobial agents can prevent drug binding, thereby conferring resistance (Li Y. et al., 2023). Fluoroquinolones specifically target the bacterial enzymes DNA gyrase and topoisomerase IV (Aldred et al., 2014). DNA gyrase is the only bacterial enzyme capable of introducing negative supercoils into DNA, a process essential for initiating DNA replication. It also facilitates replication by removing positive supercoils that accumulate ahead of the replication fork or during the transcription of specific genes. Topoisomerase IV, in the final stages of DNA replication, ensures the proper segregation of interlinked daughter chromosomes into daughter cells. Fluoroquinolones inhibit these enzymes by stabilizing the DNA–DNA gyrase or DNA–topoisomerase IV complexes (Aldred et al., 2014; Bush et al., 2020). Stabilization of the DNA–DNA gyrase complex obstructs replication fork progression, rendering previously reversible DNA-enzyme complexes irreversible. This leads to DNA damage, strand breaks, and rapid inhibition of DNA synthesis, ultimately resulting in cell death. Mutations within the quinolone resistance-determining regions of DNA gyrase and topoisomerase IV, particularly in the GyrA and ParE subunits, are primarily responsible for quinolone resistance (Huy, 2024; Li Y. et al., 2024; Wang Q. et al., 2025).

With the rising prevalence of CRKP, treatment options are increasingly limited (Chirabhundhu et al., 2024). Tigecycline, one such option, targets the 30S and 16S ribosomal subunits. By reversibly binding to these subunits, tigecycline prevents aminoacyl-tRNA from attaching to the ribosome–mRNA complex, thereby inhibiting protein synthesis (Huy, 2024; Korczak et al., 2024; Yaghoubi et al., 2022). Chromosomally mediated modifications occurring in these subunits reduce the sensitivity to tigecycline. Additionally, mutations in genes such as *tetM*, which encode proteins that protect ribosomes, also reduce sensitivity to tigecycline (Wang Q. et al., 2025; Korczak et al., 2024). The target sites for aminoglycosides are specific nucleotide residues in the 16S subunit of ribosomal RNA (rRNA). However, modification of these sites by 16S rRNA methyltransferase enzymes prevents aminoglycoside binding (Wang Q. et al., 2025). Polymyxins are cationic polypeptide antibiotics composed of five different compounds, polymyxin A through E. The clinically used forms are polymyxin E (colistin) and B. Despite its limited use due to nephrotoxicity and neurotoxicity, tigecycline has emerged as one of the few remaining therapeutic options for infections caused by CRKP and is often considered a last-resort antibiotic (Huy, 2024; Ding Y. et al., 2023). Colistin, a cationic antimicrobial, targets the negatively charged phosphate groups of the lipid A moiety within the LPS of the bacterial outer membrane. The binding of colistin to these sites disrupts membrane integrity, resulting in leakage of cellular contents and subsequent bacterial cell lysis. However, the binding of positively charged L-Ara-4N and phosphoethanolamine (PetN) molecules to the LPS site prevents the binding of cationic colistin, leading to the development of resistance. The addition of L-Ara4N and pEtN to lipid A results from mutations in the genes encoding two-component regulatory systems, such as *phoP/phoQ, pmrA/pmrB*, and *crrABC* (Ding Y. et al., 2023; Alousi et al., 2024) (Figure 2).

#### 3.2.3 β-lactamase production

β-lactam antibiotics are categorized into four main groups according to their chemical characteristics: penicillins, carbapenems, cephalosporins, and monobactams (Karami-Zarandi et al., 2023). These antimicrobials are commonly employed in clinical settings because of their broad treatment options and proven effectiveness. However, resistance to β-lactam antibiotics continues to increase rapidly worldwide. The primary reason for this is the presence of β-lactamases (Li Y. et al., 2024). β-lactamases are versatile enzymes with relatively limited structural diversity, produced by a wide range of bacterial species. Their primary function is to hydrolyse β-lactam-containing compounds. Sequence-based analyses have classified β-lactamases into four molecular groups, A, B, C, and D, based on their molecular size and active-site amino acid sequence homology (Tooke et al., 2019; Alfei and Schito, 2022). Groups A, C, and D contain a serine residue at the active site and are categorized as serine β-lactamases. In contrast, group B contains zinc ions and is classified as metallo- β-lactamases (Alfei and Schito, 2022). *K. pneumoniae* exhibits a high capacity for transferring mobile genetic elements, including transposons and insertion sequences, to plasmids. This ability facilitates the dissemination of AMR genes to other strains and even across bacterial species (Karami-Zarandi et al., 2023; Navon-Venezia et al., 2017). Among all β-lactamases, class A enzymes are the most widespread. Typical class A β-lactamases in Gram-negative bacteria include TEM, SHV, CTX-M, VEB, GES, PER, TLA, SFO, BES, BEL, and KPC. Temoneira (TEM) was the first plasmid-encoded β-lactamase, conferring activity against aminopenicillins and early-generation cephalosporins (Tooke et al., 2019; Husna et al., 2023; Castanheira et al., 2021). SHV (Sulfhydryl Variant) exhibits a similar substrate profile to TEM. Initially chromosomally encoded in *K. pneumoniae*, SHV was later found to be carried on plasmids (Tooke et al., 2019; Tsang et al., 2024). Vietnam Extended-Spectrum β-lactamase (VEB) represents a minor subgroup of class A enzymes, comprising twelve variants, and is predominantly detected in non-fermentative species such as *Pseudomonas aeruginosa* and *Acinetobacter baumannii*, as well as in *Enterobacteriaceae* (Tooke et al., 2019; Lahiri and Alm, 2016). Guiana Extended-Spectrum (GES) was first reported in *K. pneumoniae* isolates in 1998 and contributes to carbapenem resistance (Castanheira et al., 2021). KPC (*K. pneumoniae carbapenemase*) is plasmid-borne and primarily found in *K. pneumoniae* (Alfei and Schito, 2022). Pseudomonas extended resistant (PER), first isolated from *P. aeruginosa*, hydrolyses most penicillins and cephalosporins. Brazil Extended-Spectrum (BES), Belgium Extended-β-lactamase (BEL), Tlahuica Indians (TLA), and Serratia fonticola (SFO) enzymes are less common class A β-lactamases (Castanheira et al., 2021). Cefotaximase (CTX-M) has natural activity against oxyiminocephalosporins such as cefotaxime. In addition to being the β-lactamase with the most variants, it is also the most rapidly spreading enzyme worldwide. This spread rate has been named the CTX-M pandemic (Tooke et al., 2019; Cantón et al., 2012). ESBLs are hydrolytic enzymes produced by various Gram-negative bacteria within the Enterobacteriaceae family. ESBLs hydrolyse third-generation cephalosporins and aztreonam but not cephamycins, and their activity can be inhibited by β-lactamase inhibitors such as clavulanic acid, sulbactam, and tazobactam. These enzymes represent mutated variants of β-lactamases that display structural and functional modifications (Husna et al., 2023). Prominent ESBLs include TEM, SHV, CTX-M, VEB, GES, PER, TLA, SFO, BES, and BEL (Husna et al., 2023; Castanheira et al., 2021). Since penicillin resistance was first documented in *K. pneumoniae* in the 1960s, high-level resistance to most β-lactam antibiotics has developed over the past six decades, positioning *K. pneumoniae* as a significant contributor to nosocomial infections (Karami-Zarandi et al., 2023). The first ESBL identified in *K. pneumoniae* was SHV-2, followed a few years later by TEM-3. Subsequently, the CTX-M enzyme was introduced into *K. pneumoniae* via plasmid-mediated transfer, becoming the most prevalent ESBL in this species after the 1990s (Navon-Venezia et al., 2017). Concurrently, less common plasmid-mediated ESBLs, including SFO, PER, TLA, KLUC, and GES, have also been reported in *K. pneumoniae*. The bacterium's remarkable capacity to acquire antibiotic resistance has facilitated the global dissemination of ESBL-producing strains. According to WHO reports, the prevalence of ESBL-producing *K. pneumoniae* has reached 50% in many regions, with community-based resistance rates approaching 30% (Li Y. et al., 2023; Wang G. et al., 2020; Li P. et al., 2018).

As ESBL prevalence increased worldwide, carbapenems, initially imipenem, became the treatment of choice. However, extensive and uncontrolled carbapenem use led to the rapid emergence of CRKP within approximately 4 years (Karami-Zarandi et al., 2023). The genes encoding carbapenemases are primarily carried on plasmids, facilitating their horizontal dissemination. Genes responsible for carbapenem resistance, which encode carbapenemases, are classified within the β-lactamase groups of classes A, B, and D. Specifically, KPC, GES, IMI, SME, and NMCA are categorized under Class A. IMI, SME, and NMCA are chromosomally situated and are inhibited by clavulanic acid. In contrast, KPC and GES are harbored on plasmids. The most prevalent carbapenemase gene is *KPC*. The transposon Tn4401, which carries the *KPC* gene, exhibits a heightened capacity to transfer this gene to other plasmids and facilitate clonal dissemination. *KPC*-carrying strains have been reported from all continents (Huy, 2024; Navon-Venezia et al., 2017). *KPC* can inactivate penicillins, cephalosporins, and monobactams but is also resistant to carbapenems. It was first identified in the United States. Because bacteria producing the KPC enzyme can acquire resistance to many antibiotics, they contribute to increased mortality rates in immunocompromised patients infected with these bacteria (Moya and Maicas, 2020).

NDM, IMP, and VIM are class B carbapenemases. They are clinically significant carbapenemases because they hydrolyse all β-lactam antibiotics except aztreonam (Bedenić et al., 2016). Two years after the use of imipenem to treat *K. pneumoniae* infections, imipenem-resistant strains began to emerge. The first class B β-lactamase identified in *K. pneumoniae* isolates is IMP-1, which was reported from Japan (Navon-Venezia et al., 2017). NDM-1 was initially identified in a *K. pneumoniae* isolate from New Delhi. This enzyme, located on the bacterial outer membrane, sequesters zinc from the host. Vesicles may also facilitate the transport of the active enzyme NDM-1 via exocytosis. This process allows it to adhere to the membranes of other bacteria, rendering them temporarily resistant to antibiotics (Moya and Maicas, 2020; Navon-Venezia et al., 2017; Shi et al., 2019). Bacteria that carry this enzyme are called superbugs because they hydrolyse most β-lactam antibiotics, including carbapenems (Zhao et al., 2022). Class D β-lactamases are known as oxacillin or OXA class. The most clinically important OXA class β-lactamase is the OXA-48 type. This enzyme hydrolyses carbapenems, cephalosporins, and the penicillin oxacillin, from which it takes its name (Han et al., 2022; Braun et al., 2024). There are many variants of the oxa-48 type: OXA-48, OXA-162, OXA-244, OXA-204, OXA-232, and OXA-181. Plasmid-borne OXA-48 can be easily transmitted from *K. pneumoniae* to other ESKAPE pathogens and can also spread clonally with ST147, ST307, ST15, and ST14, leading to outbreaks. The global prevalence of this enzyme is due to its transport by plasmids (Pitout et al., 2019; Lerminiaux et al., 2024).

AmpC β-lactamases not only hydrolyse broad- and extended-spectrum cephalosporins but are also resistant to inhibition by β-lactamase inhibitors such as clavulanic acid, sulbactam, and tazobactam. They remain susceptible to carbapenems and fourth-generation cephalosporins. However, due to the presence of multiple gene copies, *ampC* genes can be efficiently overexpressed when carried on plasmids (Huy, 2024; Li Y. et al., 2023; Jomehzadeh et al., 2022). In *K. pneumoniae*, the coexistence of *ampC* expression with porin loss and heightened efflux contributes to carbapenem resistance. Plasmid-mediated *ampC* genes identified in *K. pneumoniae* include CIT, DHA, FOX, MOX, EBC, and ACC. These plasmid-borne AmpC enzymes likely originated from the mobilization of chromosomal *AmpC* genes onto plasmids (Karami-Zarandi et al., 2023; Navon-Venezia et al., 2017; Jomehzadeh et al., 2022). The mobility of *ampC* genes between chromosomes and plasmids facilitates the rapid dissemination of resistance in clinical and environmental settings, often leading to outbreaks. In healthcare facilities, plasmid-mediated *ampC* spread has been associated with nosocomial outbreaks, complicating therapeutic management and imposing increased economic burdens (Roberts et al., 2024) (Tables 2, 3; Figure 2).

**Table 2:** Major β-lactamase classes and clinically relevant variants reported in *K. pneumoniae*.

β-lactamase class	Representative enzymes/ variants	Hydrolysed antibiotics	Genetic location/ dissemination	Inhibitor susceptibility	Clinical relevance/ epidemiology	References
Class A (Serine β-lactamases)	TEM, SHV, CTX-M, KPC, GES, VEB, PER, BES, BEL, TLA, SFO	Penicillins and cephalosporins; KPC and some GES variants also hydrolyse carbapenems	Predominantly plasmid-mediated; frequently associated with transposons and insertion sequences	Most ESBLs are inhibited by clavulanic acid, sulbactam, and tazobactam; KPC shows reduced susceptibility	Largest β-lactamase family; includes major ESBLs and KPC carbapenemase CTX-M is responsible for the global “CTX-M pandemic”	Bush et al., 2020; Wang Q. et al., 2025; Chirabhundhu et al., 2024; Korczak et al., 2024; Yaghoubi et al., 2022; Alousi et al., 2024; Tooke et al., 2019; Alfei and Schito, 2022
Class B (Metallo-β-lactamases)	NDM, IMP, VIM	Nearly all β-lactams except aztreonam	Mainly plasmid-mediated	Not inhibited by clavulanic acid, sulbactam, or tazobactam	Major carbapenemases associated with multidrug-resistant strains and healthcare outbreaks	Liao et al., 2022; Chirabhundhu et al., 2024; Castanheira et al., 2021; Tsang et al., 2024; Lahiri and Alm, 2016
Class C (AmpC β-lactamases)	CIT, DHA, FOX, MOX, EBC, ACC	Broad- and extended-spectrum cephalosporins	Chromosomal or plasmid-mediated; plasmid amplification enhances expression	Resistant to classical β-lactamase inhibitors; generally susceptible to carbapenems	Contributes to carbapenem resistance when combined with porin loss and efflux; associated with nosocomial outbreaks	Merciecca et al., 2022; Liao et al., 2020; Farzand et al., 2021; Chirabhundhu et al., 2024; Li L. et al., 2018; Bedenić et al., 2016
Class D (OXA β-lactamases)	OXA-48, OXA-162, OXA-181, OXA-204, OXA-232, OXA-244	Carbapenems, oxacillin, and some cephalosporins	Mainly plasmid-mediated; disseminated through epidemic clones	Variable inhibitor susceptibility	Major driver of global dissemination of carbapenem-resistant *K. pneumoniae*	Han et al., 2022; Meuskens et al., 2019; Chirabhundhu et al., 2024; Cantón et al., 2012; Wang G. et al., 2020

ESBL, extended-spectrum β-lactamase.

**Table 3:** Clinically important β-lactamases identified in *K. pneumoniae*.

Enzyme	Class	First description/origin	Main resistance profile	Current clinical importance	References
TEM	A	First plasmid-mediated β-lactamase	Aminopenicillins and early cephalosporins	Prototype ESBL ancestor	Bush et al., 2020; Korczak et al., 2024; Yaghoubi et al., 2022
SHV	A	Originally chromosomal in *K. pneumoniae*	Penicillins and cephalosporins	First ESBL reported in *K. pneumoniae* (SHV-2)	Bush et al., 2020; Chirabhundhu et al., 2024; Alousi et al., 2024
CTX-M	A	Mobilized onto plasmids	Third-generation cephalosporins	Most prevalent ESBL worldwide; “CTX-M pandemic”	Bush et al., 2020; Chirabhundhu et al., 2024; Korczak et al., 2024; Alfei and Schito, 2022
VEB	A	Initially reported in Southeast Asia	Extended-spectrum cephalosporins	Emerging ESBL	Bush et al., 2020; Korczak et al., 2024; Tooke et al., 2019
GES	A	First identified in 1998	ESBL activity; some variants hydrolyse carbapenems	Emerging carbapenemase	Korczak et al., 2024; Yaghoubi et al., 2022; Alousi et al., 2024
PER	A	First isolated from *Pseudomonas aeruginosa*	Penicillins and cephalosporins	Less common ESBL	Korczak et al., 2024; Yaghoubi et al., 2022
BES	A	Rare enzyme	Extended-spectrum β-lactams	Uncommon ESBL	Yaghoubi et al., 2022
BEL	A	Rare enzyme	Extended-spectrum β-lactams	Uncommon ESBL	Yaghoubi et al., 2022
TLA	A	Rare enzyme	Extended-spectrum β-lactams	Uncommon ESBL	Korczak et al., 2024; Yaghoubi et al., 2022
SFO	A	Originally identified in *Serratia fonticola*	Cephalosporins	Occasionally detected in *K. pneumoniae*	Korczak et al., 2024; Yaghoubi et al., 2022
KPC	A	First identified in the United States	Carbapenems, penicillins and cephalosporins	Most prevalent class A carbapenemase worldwide	Merciecca et al., 2022; Liao et al., 2022; Wang Q. et al., 2025; Chirabhundhu et al., 2024
IMP	B	First identified in Japan	Carbapenems	First MBL reported in *K. pneumoniae*	Chirabhundhu et al., 2024; Castanheira et al., 2021
VIM	B	Europe	Carbapenems	Clinically important MBL	Castanheira et al., 2021
NDM	B	First reported in New Delhi	Nearly all β-lactams except aztreonam	Globally disseminated multidrug resistance determinant	Liao et al., 2022; Chirabhundhu et al., 2024; Castanheira et al., 2021; Tsang et al., 2024; Lahiri and Alm, 2016
OXA-48	D	First described in Türkiye	Carbapenems and oxacillin	Predominant OXA carbapenemase in *K. pneumoniae*	Han et al., 2022; Meuskens et al., 2019; Chirabhundhu et al., 2024; Cantón et al., 2012; Wang G. et al., 2020
AmpC (CIT, DHA, FOX, MOX, EBC, ACC)	C	Mobilized from chromosomal AmpC genes	Broad-spectrum cephalosporins	Important mechanism of cephalosporin resistance and hospital outbreaks	Merciecca et al., 2022; Liao et al., 2020; Farzand et al., 2021; Chirabhundhu et al., 2024; Li L. et al., 2018; Bedenić et al., 2016

ESBL, extended-spectrum β-lactamase; MBL, metallo-β-lactamase.

#### 3.2.4 Aminoglycoside resistance

Aminoglycosides were commonly employed to treat bacterial infections until the 1980s, when their use declined in favor of third-generation cephalosporins, carbapenems, and fluoroquinolones (Li Y. et al., 2023). *K. pneumoniae* has since developed resistance to multiple antimicrobial classes. The overuse of agents such as aminoglycosides and fluoroquinolones has contributed to the emergence of resistant clinical *K. pneumoniae* strains, thereby increasing morbidity and mortality (Swedan et al., 2024). Aminoglycosides inhibit bacterial protein synthesis, resulting in cell death.

*K. pneumoniae* employs several mechanisms to resist aminoglycosides. One major mechanism is enzymatic modification of the drug through aminoglycoside-modifying enzymes, including O-adenyltransferases, N-acetyltransferases, and O-phosphotransferases. Genes encoding these enzymes in *K. pneumoniae* include *aac(3*′*)-II, aac(6*′*)-II, aac(6*′*)-Ib, ant(3*′*)-I*, and *aph(3*′*)-VI* (Ahmed et al., 2023). These genes are commonly acquired via plasmids and other mobile genetic elements such as transposons (Papa-Ezdra et al., 2024; El-Badawy et al., 2017).

Another mechanism involves 16S rRNA methyltransferases, which methylate specific nucleotides in the 16S rRNA subunit. This modification alters the aminoglycoside binding sites, reducing drug affinity. Genes encoding these methyltransferases include *rmtA, rmtB, rmtC, rmtD, rmtE, rmtF, rmtG, rmtH*, and *npmA*. *K. pneumoniae* isolates that have acquired these plasmid-borne genes are resistant to nearly all aminoglycosides (Sabtcheva et al., 2024). Another mechanism of aminoglycoside resistance is changes in outer membrane permeability, reduced inner membrane transport, and decreased intracellular aminoglycoside accumulation through active efflux (Li Y. et al., 2023; Ahmed et al., 2023).

Plasmids harboring 16S rRNA methyltransferase genes frequently co-carry β-lactamase genes, contributing to the emergence of MDR *K. pneumoniae* strains. The *armA* gene was initially identified on plasmid pIP1204 in *K. pneumoniae* and on plasmid pCTX-M3 in Citrobacter freundii, both of which also contained the ESBL gene *bla*_CTX − M−3_. In *K. pneumoniae* isolates from Italy and China, *armA* was found in conjunction with *bla*_KPC − 2_. Similarly, clinical isolates from India and Switzerland were reported to carry both *bla*_NDM − 1_ and *rmtA* on the same plasmid (Poirel et al., 2011).

Other documented instances include a *K. pneumoniae* isolate from Nepal harboring *rmtC* and *bla*_NDM − 1_ (Tada et al., 2013), a Brazilian isolate carrying *rmtD* with *bla*_KPC − 2_ (Bueno et al., 2013), a strain from La Réunion Island containing both *rmtF* and *bla*_NDM − 1_ (Galimand et al., 2012), and a Brazilian clinical isolate producing *KPC* along with *rmtG* (Bueno et al., 2013). The co-occurrence of 16S rRNA methyltransferase genes with carbapenemase genes critically reduces the already limited therapeutic options for MDR and extensively drug-resistant (XDR) *K. pneumoniae* infections (Doi et al., 2016) (Figure 2).

#### 3.2.5 Colistin resistance

The management of infections caused by CRKP is challenging, with high associated morbidity and mortality. This situation has necessitated the use of colistin as a last-resort antibiotic. However, the increased use of colistin in both human and veterinary medicine has rapidly driven the emergence of colistin-resistant strains (Gharaibeh et al., 2022). Colistin resistance is acquired through chromosomal mutations or plasmid transfer (Aghapour et al., 2019). The primary target of colistin is the LipidA moiety of the LPS molecule (Velkov et al., 2013; Gurjar, 2015). Therefore, modifications and mutations in the LPS molecule through various means lead to resistance. These can include decreased affinity for colistin due to a reduction in the negative charge of LPS, overexpression of efflux pump systems, and overproduction of capsule polysaccharides (Aghapour et al., 2019).

Plasmid-borne *mcr* genes have been horizontally transferred to *K. pneumoniae*. These genes belong to the pEtN transferase family and confer colistin resistance by catalyzing the addition of pEtN to Lipid A. *K. pneumoniae* strains harboring *mcr* acquire colistin resistance independently of other mechanisms, typically resulting in a four- to eight-fold increase in colistin minimum inhibitory concentration (MIC) (Liu et al., 2016). To date, 10 *mcr* variants (*mcr-1* to *mcr-10*) have been described (Nang et al., 2019).

The *mcr-1* gene was initially identified in an *E. coli* isolate in China (Liu et al., 2016) and subsequently detected in *K. pneumoniae*, other *Enterobacteriaceae, Pseudomonas spp., Aeromonas spp*., and *Acinetobacter lwoffii*. In *K. pneumoniae*, additional *mcr* variants reported include *mcr-7, mcr-8*, and *mcr-9* (Phetburom et al., 2021). The *mcr* gene has been documented in 47 countries across six continents, with its prevalence in *K. pneumoniae* ranking third after *E. coli* and *Salmonella enterica* (Nang et al., 2019). CRKP is associated with mortality rates exceeding 50%, particularly in ICUs and bloodstream infections, and several studies have reported the coexistence of *mcr-1, mcr-7*, and *mcr-8* in these strains (Gharaibeh et al., 2022) (Figure 2).

### 3.3 Multidrug-resistant and extensively drug-resistant strains

The global emergence of antimicrobial resistance, driven by the widespread and uncontrolled use of antimicrobial agents, has been recognized as a critical public health challenge. Among resistant pathogens, MDR strains, defined as resistant to at least one agent in three or more antimicrobial classes, and XDR strains, susceptible to only one or two antimicrobial classes, are becoming increasingly prevalent (Li L. et al., 2018; Alkofide et al., 2020).

*K. pneumoniae*, a commensal of the gastrointestinal tract in humans and animals, is an opportunistic pathogen and a major contributor to nosocomial infections, accounting for approximately one-third of Gram-negative hospital-acquired infections. This bacterium demonstrates a remarkable capacity to acquire and disseminate antimicrobial resistance. It can transmit resistance genes vertically to progeny as well as horizontally to other strains, species, and genera via mobile genetic elements such as plasmids and transposons (Li L. et al., 2018).

*K. pneumoniae* strains frequently harbor multiple β-lactamase genes simultaneously. All known *bla* genes have been reported in *K. pneumoniae*, and the bacterium can acquire transposons carrying different bla genes on a single plasmid. Moreover, a single strain may carry multiple plasmids, each harboring distinct resistance determinants, further complicating treatment strategies. For example, IncA/C2 plasmids encode aminoglycoside-modifying enzymes, as well as quinolone, trimethoprim, and chloramphenicol resistance genes, along with NDM carbapenemase and AmpC β-lactamase, while pKpQIL and pKPN3 plasmids are frequently found together in the same strain (Navon-Venezia et al., 2017).

The presence of AMR genes on multiple plasmids in hospital-associated *K. pneumoniae* isolates has facilitated the emergence of highly resistant, or “super-resistant,” strains. These strains display resistance across multiple antibiotic classes. In such isolates, ESBL and carbapenemase genes often coexist with aminoglycoside-modifying enzymes, while 16S rRNA methyltransferase genes are frequently found alongside β-lactamases, including KPC and NDM. The accumulation of additional resistance mechanisms, such as porin loss, changes in outer membrane permeability, or efflux pump overexpression, can give rise to pan-drug-resistant strains, resistant not only to MDR and XDR categories but to nearly all available antimicrobial agents. Consequently, infections caused by these strains are extremely difficult to treat (Navon-Venezia et al., 2017; Bedenić et al., 2016; Zhao et al., 2022; Li L. et al., 2018).

## 4 Epidemiology and risk factors

### 4.1 Global prevalence and hotspots

*K. pneumoniae is* recognized as one of the most important global pathogens due to its ability to cause severe infections and rapidly acquire antimicrobial resistance. Initially considered an opportunistic pathogen affecting mainly hospitalized or immunocompromised patients, it has now emerged as a critical public health threat, particularly with the increasing prevalence of MDR and CRKP strains. Together with other members of the ESKAPE group, *K. pneumoniae* is a leading cause of healthcare-associated infections (HAIs) that are difficult to manage clinically (Karlowsky et al., 2022; Karampatakis et al., 2023).

According to reports from the WHO and the Centers for Disease Control and Prevention (CDC), *K. pneumoniae* is consistently ranked among the leading causes of ventilator-associated pneumonia, bloodstream infections, urinary tract infections, and sepsis in hospital settings (Han et al., 2022; Gan et al., 2025). More recently, the emergence of hvKP has reshaped the epidemiological profile of the pathogen, as these variants are capable of causing invasive infections such as liver abscesses, meningitis, and septicemia even in otherwise healthy individuals (Han et al., 2022; Karami-Zarandi et al., 2023; Li Y. et al., 2024; Karlowsky et al., 2022). Epidemiological data indicate that *K. pneumoniae* exhibits substantial geographical variation in prevalence and AMR patterns. The highest prevalence of CRKP has been reported in East and Southeast Asia, particularly in China, India, and Thailand, where excessive and often uncontrolled antibiotic use in both human medicine and veterinary practices has facilitated the selection and dissemination of resistant strains. Southern European countries, including Italy, Greece, and Spain, also represent persistent hotspots, with some tertiary care hospitals reporting CRKP rates exceeding 50% of all *K. pneumoniae* isolates. In North America, Latin America, and the Middle East, an upward trend of ESBL-producing isolates has been observed, highlighting the global expansion of AMR mechanisms. The formation of hotspots is influenced by multiple factors, including high patient density in hospitals, frequent use of invasive medical devices, gaps in infection control protocols, and inadequate antibiotic stewardship programmes, as well as international travel, medical tourism, and the global movement of food (Gan et al., 2025; Zhu et al., 2025).

The global distribution of *K. pneumoniae* is highly heterogeneous. The highest prevalence of CRKP has been documented in China, India, and several Southeast Asian countries, where unregulated antibiotic consumption in both human and veterinary medicine plays a major role in driving resistance (Ferreira et al., 2018). In Europe, Italy and Greece represent well-established hotspots, with hospital-based surveillance indicating CRKP prevalence rates exceeding 50% in certain institutions. In the United States and Latin America, a growing trend of resistance has also been reported, particularly due to the spread of ESBL-producing isolates (Hu et al., 2024).

The rapid dissemination of resistance is further fuelled by horizontal gene transfer. Resistance determinants such as *bla*_KPC_, *bla*_NDM_, *bla*_OXA − 48_, and *bla*_VIM_ have been identified in multiple regions. They are frequently carried on mobile genetic elements, enabling their rapid spread across bacterial populations and geographical boundaries (Roberts et al., 2024; Gan et al., 2025).

From an epidemiological perspective, the current situation reflects a multidimensional crisis characterized by (a) the rapid global rise of MDR strains; (b) the convergence of resistance with hypervirulence, leading to more severe clinical outcomes; (c) geographical clustering of resistance, creating global “hotspots”; and (d) limited therapeutic options accompanied by increased morbidity and mortality (Tooke et al., 2019; Hu et al., 2024).

**Figure 3:**
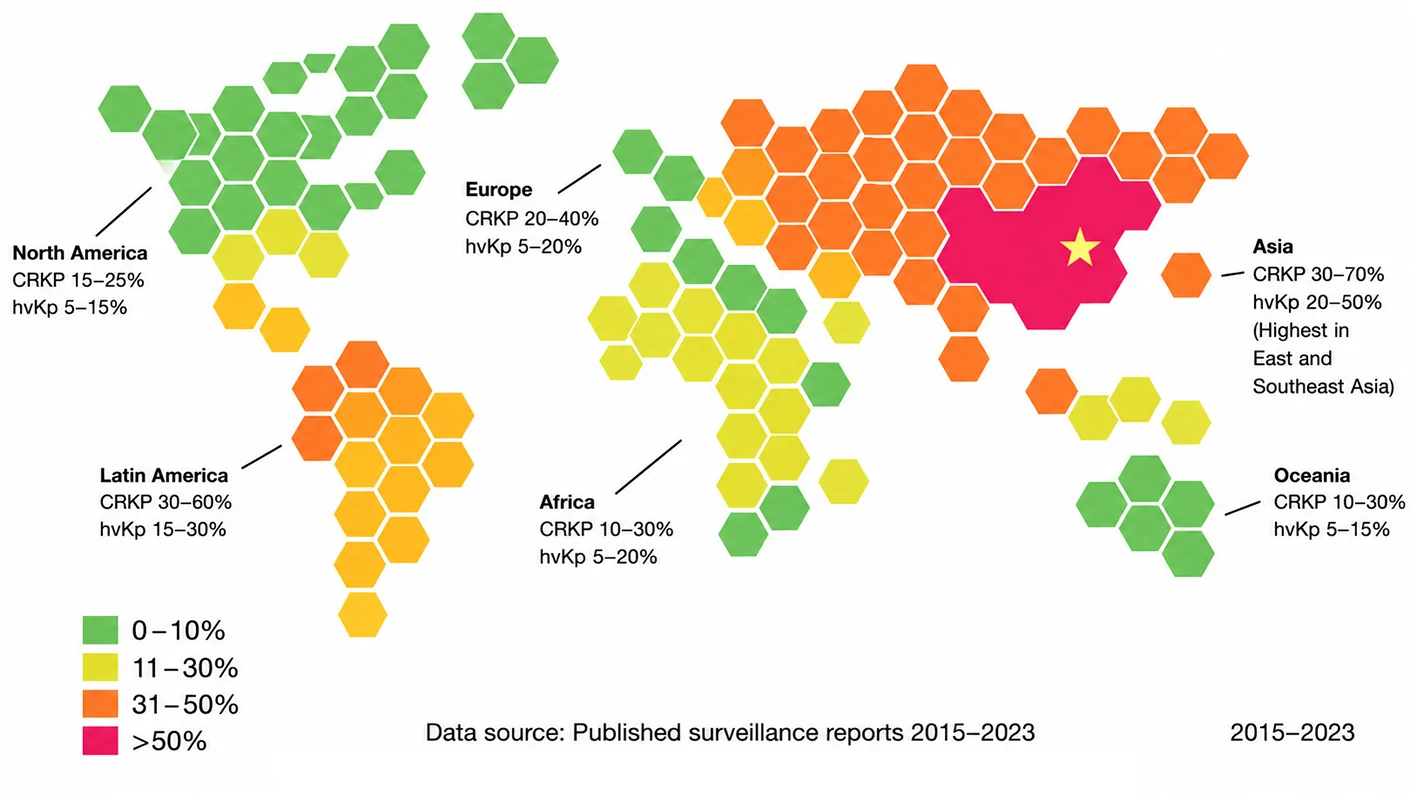
Global hotspots of carbapenem-resistant *K. pneumoniae* (CRKP). This hexagonal world map illustrates the global prevalence of CRKP. Regions with higher resistance rates are depicted in shades of red, indicating significant antimicrobial resistance hotspots such as China, India, Greece, and Italy. Moderate prevalence areas appear in yellow, while low-prevalence regions are shown in green. Star symbols denote countries where hvKP have been reported. This visualization highlights the uneven but alarming distribution of CRKP across continents, emphasizing the need for coordinated international surveillance and containment strategies. Data Source: Published surveillance reports 2015–2023. CRKP, carbapenem-resistant K. pneumoniae; hvKP, hypervirulent *K. pneumoniae*.

Importantly, the epidemiology of *K. pneumoniae* extends beyond the clinical domain and is strongly influenced by patterns of antibiotic consumption, international patient transfer, medical tourism, migration, and the global trade of food and livestock products. These factors have facilitated the transition of *K. pneumoniae* from a regional concern to a truly global threat (Phetburom et al., 2021). Overall, available evidence underscores the urgent need for coordinated international surveillance, stringent infection control measures, and prudent antibiotic stewardship programmes. Without such interventions, the continuing rise of drug-resistant *K. pneumoniae* strains may lead to further limitations in treatment options and escalating challenges for global public health (Karlowsky et al., 2022; Karampatakis et al., 2023) (Figure 3).

#### 4.1.1 Temporal trends

Longitudinal surveillance studies over the past decade have demonstrated a steady increase in MDR and CRKP isolates worldwide. For example, in several hospitals in China and India, the prevalence of CRKP has doubled over the last 10 years, while Europe has experienced sporadic but severe outbreaks associated with nosocomial transmission. Data from the United States and Brazil indicate an increasing trend in ESBL-producing *K. pneumoniae*, suggesting that resistance mechanisms are spreading beyond traditional hotspots. These trends are particularly concerning since they correlate with higher morbidity, mortality, and healthcare costs, and limit the effectiveness of standard empiric therapies (Gan et al., 2025; Wyres and Holt, 2018; Cantón et al., 2023).

#### 4.1.2 Molecular and genetic determinants of resistance

The global dissemination of MDR and CRKP is primarily driven by horizontal gene transfer through plasmids, transposons, and integrons. Key resistance genes include *bla*_KPC_, *bla*_NDM_, *bla*_OXA − 48_, and *bla*_VIM_, which confer resistance to carbapenems, and *bla*_CTX − M_, and *bla*_SHV_, responsible for extended-spectrum β-lactamase production. The presence of mobile genetic elements allows these genes to spread rapidly between bacterial populations and across geographical boundaries, resulting in the emergence of novel resistant clones. Biofilm formation on medical devices, such as catheters and ventilators, further enhances bacterial persistence and reduces antibiotic efficacy (Karlowsky et al., 2022; Cantón et al., 2023).

#### 4.1.3 Emergence of hvKP

Beyond antimicrobial resistance, the rise of hvKP has added a new layer of epidemiological concern. First described in East Asia, hvKP strains can cause severe invasive infections such as liver abscesses, meningitis, endophthalmitis, and necrotising fasciitis, often in healthy individuals with no prior healthcare exposure. HvKP strains are characterized by virulence factors including hypermucoviscosity, increased siderophore production, and enhanced capsule formation, which facilitate immune evasion and rapid tissue invasion. Alarmingly, hybrid strains combining hypervirulence and MDR have been reported in several countries, posing unprecedented challenges for both clinical management and public health interventions (Gan et al., 2025).

### 4.2 Healthcare-associated vs. community-acquired infections

Infections caused by *K. pneumoniae* are commonly classified into HAIs and community-acquired infections (CAIs), each presenting distinct epidemiological, clinical, and microbiological characteristics. Understanding these differences is crucial for both prevention and treatment strategies (Ghotaslou et al., 2007).

#### 4.2.1 Healthcare-associated infections

*K. pneumoniae* is a major contributor to HAIs, especially among patients in ICUs, those undergoing invasive procedures, or individuals with chronic comorbidities. Typical clinical manifestations include ventilator-associated pneumonia, catheter-related bloodstream infections, and urinary tract infections linked to indwelling devices (Li L. et al., 2023; Ghotaslou et al., 2007). A key challenge in HAIs is the prevalence of MDR and CRKP. These strains frequently carry genes such as *bla*_KPC_, *bla*_NDM_, *bla*_OXA − 48_, and *bla*_VIM_, which confer resistance to multiple antibiotic classes. Horizontal gene transfer via plasmids and transposons facilitates the rapid dissemination of these resistance determinants within healthcare settings. Additionally, biofilm formation on medical devices further enhances bacterial persistence and decreases antibiotic efficacy. Collectively, these factors result in limited treatment options, prolonged hospital stays, and elevated morbidity and mortality rates (Li L. et al., 2023; Meatherall et al., 2009).

#### 4.2.2 Community-acquired infections

In contrast, CAIs are often associated with hvKP strains, which, although usually more susceptible to antibiotics than HAI strains, exhibit enhanced virulence traits. These include overproduction of siderophores, hypermucoviscous capsule formation, and secretion of various toxins that facilitate tissue invasion and immune evasion. Clinically, hvKP can cause severe infections such as pyogenic liver abscesses, meningitis, endophthalmitis, and necrotising fasciitis, often in healthy adults with no prior healthcare exposure (Jung et al., 2012). Recent evidence highlights the emergence of hybrid strains that combine MDR with hypervirulence, posing an unprecedented threat to public health. Such strains have the potential to cause severe community-onset infections that are difficult to treat, bridging the gap between traditional HAIs and CAIs. In summary, the epidemiological distinction between HAIs and CAIs reflects a dual threat posed by *K. pneumoniae*: resistance-driven complications in healthcare settings and virulence-driven invasive infections in the community. Effective management requires targeted infection control measures, judicious antibiotic stewardship, and continuous surveillance to monitor the emergence and spread of both MDR and hvKP (Meatherall et al., 2009).

### 4.3 High-risk populations

Certain populations are particularly vulnerable to infections caused by *K. pneumoniae*, due to either compromised immunity or increased exposure to healthcare settings. Identifying these high-risk groups is essential for targeted prevention, early diagnosis, and effective treatment strategies (Agyeman et al., 2020).

#### 4.3.1 Immunocompromised individuals

Patients with weakened immune systems are at elevated risk of severe *K. pneumoniae* infections. This group includes individuals with malignancies, those undergoing chemotherapy, organ transplant recipients on immunosuppressive therapy, patients with HIV/AIDS, and individuals with diabetes mellitus. In these patients, infections can progress rapidly, and the presence of MDR strains significantly limits therapeutic options (Bai et al., 2023).

#### 4.3.2 ICU patients

ICU patients are highly susceptible to *K. pneumoniae* due to prolonged hospitalization, invasive medical devices (e.g., central venous catheters, mechanical ventilators), and frequent exposure to broad-spectrum antibiotics. These conditions not only increase colonization and infection risk but also facilitate the spread of MDR strains within the ICU environment (Jin et al., 2021).

#### 4.3.3 Neonates and the elderly

Newborns and elderly individuals represent additional high-risk groups. In neonates, immature immune responses render them vulnerable to bloodstream infections and sepsis. Elderly patients, often with multiple comorbidities and decreased immune competence, are at heightened risk for severe infections and complications (Zhong et al., 2025).

#### 4.3.4 Other contributing factors

Additional risk factors include prolonged or repeated antibiotic exposure, invasive surgical procedures, and coexisting chronic conditions such as chronic kidney disease or chronic lung disease. These factors may increase susceptibility to colonization and infection with both MDR and hvKP (Davoudabadi et al., 2023).

#### 4.3.5 Clinical implications

High-risk populations often experience more severe disease manifestations, longer hospital stays, and higher mortality rates. Consequently, early identification, enhanced infection control protocols, and personalized therapeutic approaches are critical to improving outcomes. Preventive strategies, including routine surveillance cultures, strict hand hygiene, and judicious antibiotic use, are particularly important in these vulnerable groups (Ni et al., 2025).

## 5 Clinical and public health challenges

Research on antibiotic resistance in *K. pneumoniae* has revealed that gaps between laboratory and clinical practice, resource limitations, and difficulties in infection control significantly limit treatment success (Agyeman et al., 2020).

### 5.1 Diagnostic difficulties (detection of resistance genes)

The accurate and rapid detection of antibiotic resistance in *K. pneumoniae* plays a critical role in reducing mortality, but current diagnostic methods face various limitations. Conventional phenotypic methods may underestimate the level of resistance, particularly in cases where multiple mechanisms are present, such as low-level carbapenemases, porin loss, and efflux pumps, leading to delayed or incorrect treatment in clinical practice (Bai et al., 2023; Jin et al., 2021). The presence of hypervirulent and carbapenem-resistant subtypes can indicate resistance evolution during treatment and further reduce the sensitivity of standard tests (Jin et al., 2021; Zhong et al., 2025). In addition, the transfer of multiple resistance genes via plasmids and their capacity for horizontal transfer leads to the emergence of heterogeneous subpopulations with different resistance profiles in the same patient, creating additional diagnostic challenges (Davoudabadi et al., 2023).

In recent years, the proliferation of molecular-based methods has partially filled these gaps, but they too have limitations. While PCR and qPCR panels can rapidly detect known genes, the emergence of new β-lactamase variants (e.g., numerous subtypes defined in the KPC or NDM families) can result in false negatives by exceeding the coverage of existing kits (Ni et al., 2025; Vidal-García et al., 2023). In this regard, rapid nanopore sequencing-based approaches show promising results in detecting β-lactamase directly from blood cultures, but standardization and cost barriers persist in routine practice (Vidal-García et al., 2023). Similarly, innovative platforms such as MALDI-TOF MS are being tested for determining colistin resistance (Germ and Pirs, 2025). In contrast, the use of biomarkers such as peg-344, iroB, iucA, and rmpA/rmpA2 beyond the traditional thread test is recommended for the reliable diagnosis of hvKP (Zhong et al., 2025). Recent studies indicate that the use of these molecular markers in conjunction with phenotypic methods can significantly improve diagnostic accuracy (Vidal-García et al., 2023; Germ and Pirs, 2025).

### 5.2 Limited treatment options

*K. pneumoniae* strains, particularly CRKP and MDR/XDR strains, are rapidly depleting treatment options in antibiotic therapy. Although carbapenems have been among the most effective agents for many years, the recent spread of carbapenemase enzymes such as KPC, NDM, VIM, and OXA-48 has largely eliminated the effectiveness of this class (Van Giang et al., 2025; Bai et al., 2022). When carbapenem resistance emerges, treatment generally shifts to tigecycline, colistin, or aminoglycosides; however, the high rate of resistance encountered with these agents also limits their effective use (Agyeman et al., 2020; Durdu et al., 2019). For example, it has been reported that colistin resistance exceeds 20% in some regions, while resistance rates for aminoglycosides and fluoroquinolones reach 50%−70%. This situation significantly limits the practical use of traditional antibiotic options due to resistance (Arato et al., 2021).

Furthermore, the resistance mechanisms of existing agents are not limited to a single class but develop in the form of cross-resistance. Since carbapenem resistance is often accompanied by fluoroquinolone and aminoglycoside resistance, the risk of treatment failure is further increased (Agyeman et al., 2020; Bai et al., 2022). Meta-analyses of CRKP infections have shown that mortality is high in patients treated with monotherapy, while combination therapies yield somewhat better results (Agyeman et al., 2020). However, the emergence of resistant subpopulations, even in combination therapies, limits the clinical response. Regional resistance differences also pose a significant problem: for example, while resistance to broad-spectrum β-lactams and aminoglycosides is reported to be over 80% in Southeast Asia and the Middle East, this rate is around 40%−50% in some European countries (Van Giang et al., 2025; Durdu et al., 2019; Arato et al., 2021). All of this indicates that the rapid spread of antibiotic resistance has severely limited current treatment options and left clinicians facing significant difficulties in determining an effective regimen (Van Giang et al., 2025).

### 5.3 Economic and healthcare system burden

The increasing antibiotic resistance of *K. pneumoniae* seriously affects not only the success of clinical treatment but also healthcare systems and the economy. Resistant infections directly increase treatment costs due to longer hospital stays, increased need for ICUs, and the necessity of isolation measures (Van Giang et al., 2025; Durdu et al., 2019). The toxicity profiles of “rescue” antibiotics such as tigecycline and colistin necessitate additional care and treatment requirements; this increases not only drug costs but also total healthcare expenditures per patient (Agyeman et al., 2020; Bai et al., 2022). Moreover, the high costs and limited availability of new-generation β-lactam/β-lactamase inhibitor combinations exacerbate treatment inequalities, particularly in low- and middle-income countries (Arato et al., 2021). According to WHO data, bacterial infections associated with antibiotic resistance caused 1.27 million direct deaths worldwide in 2019, with millions more deaths occurring indirectly; a significant portion of these were caused by ESKAPE pathogens such as *K. pneumoniae* (World Health Organization, 2022a; Murray et al., 2022). Regional analyses indicate that resistance rates to agents such as cefoxitin, doxycycline, piperacillin, and trimethoprim-sulfamethoxazole are around 35%−36% in China, while reaching levels of 80%−90% in Egypt and Brazil (Beig et al., 2024). This heterogeneity not only affects treatment success but also varies healthcare costs globally. Data from the European Center for Disease Prevention and Control show that antibiotic-resistant bacteria caused over 870,000 infections in EU/EEA countries in 2019 and imposed billions of euros in additional costs on healthcare systems (Cassini et al., 2019). The latest global analyses show that infections linked to antibiotic resistance are associated with millions of additional hospital days, hundreds of thousands of deaths, and billions of dollars in economic losses each year (Agyeman et al., 2020; Arato et al., 2021; Aggarwal et al., 2024). Therefore, the increasing antibiotic resistance of *K. pneumoniae* not only negatively affects patient prognosis but also poses a significant economic and social burden that threatens the sustainability of healthcare systems (Aggarwal et al., 2024).

## 6 Therapeutic approaches

Increased antibiotic resistance in *K. pneumoniae*, MDR/XDR profiles, biofilm-associated phenotypes, and hypervirulent subtypes complicate the treatment process, limiting treatment options to a narrow pool in most cases, leading to a clinical practice where single-agent regimens may be inadequate, thus favoring combination and individualized strategies more frequently (Agyeman et al., 2020). The clinical outcomes show significant heterogeneity, even within the same class, due to regional resistance patterns, pharmacokinetic/pharmacodynamic (PK/PD) limitations, and toxicity profiles (particularly of polymyxins, aminoglycosides, and high-dose tigecycline) (Arato et al., 2021). In this regard, the current approach relies on the integration of components such as rapid and accurate resistance/type determination (using molecular panels and accelerated methods), empirical selection based on local susceptibility data, timely de-escalation/escalation, accurate dose-duration optimisation, and antimicrobial stewardship, thereby shortening the time to effective treatment while reducing resistance selection and toxicity burden (Zhong et al., 2025; Rahmat Ullah et al., 2024). In addition, the dynamics of colistin heteroresistance, cross-resistance between various classes, and the evolution of resistance during treatment highlight the importance of combination decisions and close microbiological monitoring, requiring that selected regimens be individualized based on the focus (bacteremia, pneumonia, urinary tract infection, etc.) and host factors (comorbidity, renal function, ICU conditions, etc.) (Agyeman et al., 2020; Zhong et al., 2025; Rajakani et al., 2023).

### 6.1 Current treatment strategies

The primary strategy in the current treatment of *K. pneumoniae* infections is based on selecting active agents early on, evaluating combination therapies in appropriate cases, and improving treatment success through PK/PD optimisation (Agyeman et al., 2020). However, due to increasing resistance, standard monotherapy is often insufficient, and treatment generally requires the use of multiple agents, especially in high-risk patients (Arato et al., 2021). Recent studies indicate that β-lactam/β-lactamase inhibitor combinations such as ceftazidime-avibactam, meropenem-vaborbactam, and imipenem-rellebactam show promise against certain resistance profiles, but access limitations and regional resistance differences restrict their use (Russo et al., 2023; Zhong et al., 2025; Ni et al., 2025; Galani et al., 2021). In addition, colistin, tigecycline, and aminoglycosides are still used in the clinic as “last-resort” drugs; however, these agents are administered with caution due to their toxicity and pharmacokinetic disadvantages (Bai et al., 2022; Rahmat Ullah et al., 2024; Lee et al., 2016). Therefore, current treatment strategies encompass both the rational use of existing antibiotics with limited efficacy and the selective integration of newly developed agents within the framework of individualized approaches based on the clinical picture, local resistance patterns, and patient characteristics (Russo et al., 2023; Galani et al., 2021).

#### 6.1.1 Carbapenems and alternatives (Tigecycline, Colistin)

Carbapenems have long been among the most potent agents for treating *K. pneumoniae* infections, demonstrating high efficacy, particularly against strains producing ESBLs (Galani et al., 2021). However, the rapid spread of carbapenemases (KPC, NDM, OXA-48, and VIM) over the past decade has severely limited the effectiveness of this class and necessitated the individualization of treatment decisions based on the type of carbapenemase, regional resistance patterns, and *in vitro* susceptibility results (Russo et al., 2023; Bai et al., 2023). In addition to carbapenemase production, alterations in outer membrane porins (particularly OmpK35 and OmpK36) and the overexpression of efflux pumps such as AcrAB-TolC further reduce carbapenem susceptibility, contributing to complex resistance phenotypes that complicate therapeutic decision-making (Goodarzi et al., 2021; Muteeb et al., 2025; Ding et al., 2025). For carbapenemase-negative, ESBL-positive cases, PK/PD optimisations such as extended infusion or high-dose regimens of meropenem can enhance the clinical response in borderline susceptibility situations (Russo et al., 2023; Bai et al., 2023). Although carbapenems are still used in selected cases today, the high mortality rates associated with infections caused by CRKP strains indicate that treatment success is quite limited. The inadequacy of carbapenems alone, particularly in critically ill patient groups, has led to the emergence of alternative agents in clinical practice (Ni et al., 2025; Bai et al., 2022).

Tigecycline and polymyxins (colistin, polymyxin B) are the most commonly used alternative agents at this point. Although tigecycline, a broad-spectrum glycylcycline, can be used in respiratory tract, intra-abdominal, and soft tissue infections, it is a limited option in the treatment of bacteremia and sepsis due to low serum concentrations (Jin et al., 2021; Ni et al., 2025). Additionally, the US Food and Drug Administration (FDA) has issued a safety warning for tigecycline due to an increased risk of mortality, emphasizing that caution should be exercised, particularly regarding its use as monotherapy in bacteremia (FDA, 2019). One study has shown that resistance can develop rapidly under tigecycline, especially during the treatment of hypervirulent ST11 clones (Jin et al., 2021). This rapid resistance evolution has been mainly associated with the overexpression of multidrug efflux systems, particularly AcrAB-TolC and OqxAB, together with regulatory alterations involving the ramA/ramR pathway and, less frequently, mutations affecting the ribosomal target encoded by rpsJ (Shah et al., 2025; Ding et al., 2025; Lin et al., 2024). Recent studies have also highlighted collateral sensitivity between tigecycline and aminoglycosides, suggesting that carefully designed sequential or combination strategies may help suppress resistant subpopulations and delay resistance evolution (Lei et al., 2024; Ma et al., 2023). Polymyxins, particularly colistin, still play a critical role in the treatment of CRKP. However, serious side effects such as nephrotoxicity and neurotoxicity limit their use; furthermore, colistin heteroresistance makes the clinical response unpredictable. Due to the pharmacokinetic advantages of polymyxin B in systemic infections, many guidelines recommend it over colistin. Colistin/CMS, on the other hand, requires therapeutic drug monitoring (TDM) and dose adjustment based on renal function due to its pro-drug nature and high risk of nephrotoxicity (Nation et al., 2015). Therefore, new methods for reliably detecting colistin resistance have been developed in recent years, with broth microdilution being accepted as the gold standard, while disk diffusion and E-test methods have been reported to be unreliable. Furthermore, the possibility of mcr-mediated resistance and heteroresistance must be considered in clinical decisions (Germ and Pirs, 2025). Besides plasmid-mediated mcr genes, chromosomal modifications involving the PmrAB, PhoPQ, and CrrAB regulatory systems, as well as mgrB inactivation, represent major mechanisms underlying colistin resistance (Zeb et al., 2026; RoyChowdhury et al., 2025; Rodríguez-Santiago et al., 2025). To overcome the nephrotoxicity and unpredictable heteroresistance associated with colistin monotherapy, combination regimens with fosfomycin, aminoglycosides, or meropenem have been increasingly investigated to improve antibacterial activity and reduce microbiological failure in selected patients, although their clinical benefits remain dependent on the infection site, susceptibility profile, and patient characteristics (Lin et al., 2024; Koul et al., 2024).

Despite all these limitations, the careful use of carbapenems in selected cases and the individualized use of tigecycline and polymyxins with focus-specific, correct dosage and duration, while monitoring for toxicity, remain central to current treatment practice. Close microbiological monitoring and susceptibility testing are mandatory, as rapid resistance evolution can develop under these agents in hvKP/CRKP strains (Jin et al., 2021). Therefore, in current clinical practice, the role of these drugs is carefully determined, taking into account the disease focus, pharmacokinetic properties, and toxicity profiles; they are often supported by combination therapies to enhance their efficacy and limit the development of resistance (Ni et al., 2025; Germ and Pirs, 2025; Bai et al., 2022).

#### 6.1.2 Combination therapies

The inadequacy of single-agent therapies against MDR phenotypes and hvKP of *K. pneumoniae* has increased the importance of combination therapies in clinical practice. The fundamental rationale for combinations is their potential to overcome heteroresistance issues, achieve synergistic bactericidal activity by combining different mechanisms of action, and slow down the rapid evolution of resistance seen with single-agent therapy, given the high initial inoculum and the presence of biofilms (Rajakani et al., 2023; Kádár et al., 2013; Hickman et al., 2014). It has been reported that mortality rates in CRKP cases treated with combination therapy are lower compared to those with single therapy, but there is no statistically significant superiority in clinical and microbiological response rates (Watkins and Deresinski, 2015). Furthermore, most of the current evidence is based on *in vitro* and observational studies; due to the limited availability of randomized controlled data, the decision to combine therapies should be individualized based on patient profile, tumor location, and local resistance patterns (Rahmat Ullah et al., 2024; Watkins and Deresinski, 2015).

The most commonly used regimens in practice include colistin + carbapenem, fosfomycin + aminoglycoside, and ceftazidime-avibactam (CZA) + aztreonam (especially in MBL-producing strains) (Rajakani et al., 2023; Kádár et al., 2013; Hickman et al., 2014). Colistin- or carbapenem-based combinations offer partial advantages in suppressing resistance, while fosfomycin combinations can accelerate bactericidal efficacy, particularly in urinary and abdominal sites. Additionally, the CZA+aztreonam combination stands out for overcoming the resistance barrier caused by metallo-β-lactamases such as NDM/VIM/IMP (Rajakani et al., 2023; Hickman et al., 2014). This combination is particularly valuable against MBL-producing isolates because aztreonam remains intrinsically stable to metallo-β-lactamases, while avibactam protects aztreonam from co-produced ESBL and AmpC enzymes, thereby restoring its antibacterial activity. Consequently, CZA plus aztreonam has become one of the most promising therapeutic strategies for otherwise difficult-to-treat MBL-producing *K. pneumoniae* infections (Wu et al., 2019; Falcone et al., 2021; Sree et al., 2024).

Beyond conventional dual regimens, optimisation of dosing strategies and carefully selected combination partners are increasingly recognized as important approaches to maximize antibacterial efficacy while minimizing toxicity and delaying the emergence of resistance. In this context, PK/PD-guided administration and individualized combination regimens may provide additional clinical benefit, particularly in critically ill patients with a high bacterial burden or limited therapeutic options (Lin et al., 2024; Albur et al., 2015). In recent years, in addition to classic antibiotic combinations, phage–antibiotic combinations (e.g., phage+tigecycline, phage+polymyxin) have shown promising results in both *in vitro* and animal models, offering a new dimension of synergy by increasing antibiotic susceptibility. This phenomenon, commonly referred to as phage–antibiotic synergy (PAS), is thought to arise from phage-driven alterations in bacterial surface receptors and resistance-associated pathways, including efflux systems, which can increase bacterial susceptibility to previously ineffective antibiotics (Ding et al., 2025; El-Din et al., 2025). Such reciprocal interactions may not only enhance bacterial killing but also reduce the emergence of phage-resistant or antibiotic-resistant subpopulations, thereby extending the therapeutic potential of both agents (Xing et al., 2025b). Therefore, combined approaches will play an important role in the future, not only in the optimisation of existing agents but also in the integration of innovative treatment strategies (Zhu et al., 2025; Farooq et al., 2025).

### 6.2 Novel and emerging treatments

In response to increasing antibiotic resistance, the management of *K. pneumoniae* infections requires not only the optimisation of existing antibiotics but also the development of new-generation treatment strategies. In this context, β-lactam/β-lactamase inhibitor combinations and siderophore cephalosporins (e.g., cefiderocol) show promise, particularly against carbapenemase-producing strains (Zhong et al., 2025; Arato et al., 2021). Additionally, phage therapy is gaining increasing attention due to its effectiveness against resistant strains in both *in vitro* and clinical cases; indeed, personalized phage applications can suppress resistance development while also reducing virulence (Li J. et al., 2023; Hatfull et al., 2022; Strathdee et al., 2023; Zaki et al., 2023; Mohammadi et al., 2023; Singh et al., 2024). Recent advances in phage engineering, adaptive evolution, and rational phage cocktail design have expanded opportunities to increase the host range of naturally occurring phages and improve antibacterial activity against genetically diverse MDR *K. pneumoniae* isolates (Venkatesan et al., 2025). Representative lytic bacteriophages with activity against MDR *K. pneumoniae* have further strengthened the therapeutic potential of this approach and support its continued clinical development. In addition, biofilm-focused approaches and advanced antimicrobial polymer-based delivery systems are being evaluated as alternative options for improving treatment success in resistant phenotypes (de Oliveira et al., 2025; Lou et al., 2018). Lastly, recent studies on antimicrobial peptides and phage–antibiotic combinations (e.g., phage+tigecycline, phage+polymyxin) are opening a new window of synergy in the treatment of resistant *K. pneumoniae* infections (Zhu et al., 2025; Farooq et al., 2025; Mwangi et al., 2025). Furthermore, emerging technologies—including antimicrobial nanoparticle platforms, antisense oligonucleotides, and CRISPR-Cas-based gene editing strategies—are expanding the therapeutic landscape by directly targeting bacterial resistance mechanisms and may provide complementary approaches for the management of MDR infections (Li Y. et al., 2024; Muteeb et al., 2025; Feng and Yin, 2026).

#### 6.2.1 New β-lactam/ β-lactamase inhibitors

One of the most critical aspects of antibiotic resistance is carbapenemase-producing *K. pneumoniae* isolates, and this situation has become one of the greatest threats in the management of infectious diseases over the past decade (Galani et al., 2021). Therefore, the new generation of β-lactam/β-lactamase inhibitor combinations (BL/BLI) developed against phenotypes resistant to classic agents has been one of the most important innovations that have fundamentally transformed the treatment paradigm (Zhong et al., 2025). The three primary BL/BLI agents entering clinical use, ceftazidime/avibactam (CZA), meropenem/vaborbactam (MVB), and imipenem/relebactam (I-R), stand out with their specific efficacy profiles against different carbapenemase classes (Rahmat Ullah et al., 2024; Lou et al., 2018; Yu J. et al., 2025).

CZA is used as a “rescue” agent across a broad clinical spectrum, from resistant bacteremias to complicated intra-abdominal infections, due to its potent activity against Ambler class A (particularly KPC) and D (OXA-48-like) enzymes (Yu J. et al., 2025). MVB exhibits high efficacy due to vaborbactam's potent inhibitory effect on class A β-lactamases (KPC); however, it does not restore meropenem activity in isolates producing class B (MBL: NDM, VIM, IMP) and class D (OXA-48) β-lactamases (Lee et al., 2016). I-R, on the other hand, provides a meaningful gain in resistance caused by class A (KPC) and AmpC (C) in practice, and is among the first options considered for CRKP management along with CZA and MVB. The common ground among these three agents is that success is determined by rapid carbapenemase classification, accurate susceptibility testing, and individualized patient management, supported by appropriate resource control (Zhong et al., 2025).

Furthermore, the efficacy of existing BL/BLI agents is limited in strains producing MBL (NDM, VIM, and IMP), and this resistance gap highlights the developing aztreonam/avibactam (AZA) combination. AZA, which has not yet entered widespread clinical use, is one of the most promising candidates against MBL-producing isolates, and the CZA+aztreonam combination is being used as a temporary strategy in some centers during this process (Lee et al., 2016). However, resistance mechanisms that can rapidly develop under selective pressure, particularly against CZA and other BL/BLI agents, such as KPC variants, porin loss, and PBP target changes, are prompting clinicians to implement close microbiological monitoring, early regimen adjustments, and combination strategies when necessary (Rahmat Ullah et al., 2024). Among these mechanisms, mutations within the blaKPC gene, particularly those affecting the Ω-loop region, have emerged as an important cause of CZA resistance by reducing the inhibitory activity of avibactam while preserving ceftazidime hydrolysis (Tooke et al., 2023; Ding L. et al., 2023). Interestingly, these structural alterations may partially restore carbapenem susceptibility, creating opportunities for salvage treatment with carbapenem-based BL/BLI combinations such as meropenem-vaborbactam or imipenem-relebactam in carefully selected cases (Lei et al., 2024; Tiseo et al., 2021). This evolving resistance profile further emphasizes the importance of continuous microbiological surveillance and individualized therapeutic decision-making throughout treatment (Lei et al., 2024; Gato et al., 2023).

The global spread of OXA-48-like enzymes and the possibility of multiple carbapenemases being present in the same isolate further emphasize the importance of rapid molecular testing in the initial choice of treatment. Several next-generation β-lactamase inhibitors are currently under clinical development to address the therapeutic limitations associated with MBL-producing isolates (Wu et al., 2019). In addition to aztreonam-avibactam, novel boronate-based inhibitors and β-lactam enhancers, including nacubactam and zidebactam, have demonstrated encouraging activity by restoring β-lactam efficacy against resistant Gram-negative pathogens and may further expand future treatment options for MDR *K. pneumoniae* infections (Moya et al., 2017; Monogue et al., 2018). In summary, the rational and targeted use of new-generation BL/BLI agents is positioned as one of the most strategic tools for treating resistant *K. pneumoniae*, both today and in the future (Rajakani et al., 2023).

#### 6.2.2 Phage therapy

Phage therapy is a highly host-specific, bactericidal biotherapeutic option that is regaining strength against ESKAPE pathogens such as *K. pneumoniae* during a period of rising antibiotic resistance (Li L. et al., 2024). Recent comprehensive reviews emphasize that phage therapy is based on the principles of increasing target specificity through personalized/cocktail approaches, excluding lysogenic (integrating) phages and selecting only lytic phages through genomic characterization, and integrating complementary strategies to support efficacy in biofilm and high inoculum conditions (Zhong et al., 2025; Li J. et al., 2023). Recent studies on *K. pneumoniae* also highlight the importance of selecting an appropriate phage panel, optimizing the MOI, and conducting susceptibility testing and dynamic monitoring during treatment due to the host range varying according to capsule types and clonal lineages (Xing et al., 2025b; Mohammadi et al., 2023; Singh et al., 2024; Xing et al., 2025a). Recent studies have increasingly focused on phage engineering, adaptive evolution, and the development of rational phage cocktails to expand the host range of naturally occurring phages and broaden antibacterial activity against genetically diverse MDR *K. pneumoniae* isolates. These approaches may improve therapeutic coverage while reducing the likelihood of treatment failure associated with narrow host specificity (Xing et al., 2025a,b; Ghatbale et al., 2025; Burke et al., 2025).

Clinical and experimental evidence is increasingly strong: In a case of MDR *K. pneumoniae* treated with personalized phage therapy, it has been shown that the development of phage resistance is associated with a simultaneous loss of virulence (Li J. et al., 2023). Similarly, experimental findings indicate that phages may not only reduce bacterial load but may also be associated with decreased virulence and increased antimicrobial susceptibility (Yu Y. et al., 2025). Preclinical and animal model data have demonstrated that phage + antibiotic combinations (e.g., phage + tigecycline, phage + polymyxin B) can provide *in vitro* and *in vivo* synergy, thereby reducing both the bacterial load and the risk of rapid resistance evolution (Zhu et al., 2025; Farooq et al., 2025). Representative lytic bacteriophages, including vB_Kpn_FOPMU1 and vB_KpnP_ZX1, have demonstrated potent inhibitory activity against MDR *K. pneumoniae* isolates, either as monotherapy or in combination with antibiotics (El-Din et al., 2025; Li P. et al., 2025; Li et al., 2022). In experimental infection models, these phages enhanced bacterial clearance while limiting the emergence of resistant bacterial subpopulations, further supporting the therapeutic potential of personalized phage-based approaches (El-Din et al., 2025; Li P. et al., 2025; Qin et al., 2024). Additionally, a recent study has identified a new *K. pneumoniae* phage (HZJ33) and reported its therapeutic potential in a CRKP infection model (Lu et al., 2025). These results support the rational use of phage therapy as a complementary strategy, particularly in clinical situations where treatment options are limited in the context of hvKP/CRKP (Zhong et al., 2025; Yu J. et al., 2025).

The efficacy of phage therapy depends on appropriate phage matching, cocktail use, sequential or rotational approaches, and smart combination decisions with antibiotics when necessary (Hatfull et al., 2022; Strathdee et al., 2023; Xing et al., 2025a). Although bacterial resistance to phages may develop through alterations in capsule biosynthesis or other surface receptors, these adaptive changes are frequently accompanied by reduced bacterial fitness, attenuation of virulence, and increased susceptibility to host immune responses or conventional antibiotics (Xing et al., 2025b; Li P. et al., 2025; Tang et al., 2023). This evolutionary trade-off represents one of the major therapeutic advantages of phage-based treatment and further supports its integration with antimicrobial therapy (Xing et al., 2025b; Le Bris et al., 2025). In addition, phage-derived enzymes, particularly depolymerases and endolysins, have emerged as promising adjunctive therapeutics because of their ability to degrade capsular polysaccharides and biofilms, thereby facilitating antibiotic penetration and improving bacterial eradication (Le Bris et al., 2025; Zhang Y. et al., 2025). During the treatment process, anticipating the potential emergence of phage resistance, close microbiological monitoring and rapid susceptibility testing should be performed; pharmacological and immunological factors such as the immune system's neutralizing antibody response should be considered (Singh et al., 2024; Xing et al., 2025a). For safety and production, Good Manufacturing Practice (GMP)-compliant manufacturing, endotoxin/pyrogen controls, and stability/shelf life are critical quality requirements (Hatfull et al., 2022; Strathdee et al., 2023). Despite the fact that the current evidence is largely based on case reports, small series, and experimental studies, experiences with compassionate use and ongoing clinical trials indicate that phage therapy may be selectively applied, particularly in cases where treatment options are exhausted or in high-risk infections (Xing et al., 2025b; Hatfull et al., 2022; Strathdee et al., 2023; Singh et al., 2024; Venkatesan et al., 2025; Xing et al., 2025a).

#### 6.2.3 Antimicrobial peptides

Antimicrobial peptides (AMPs) are evolutionarily conserved components of innate immunity, characterized by rapid bactericidal effects, biofilm-disrupting activity, and multiple modes of action distinct from classical antibiotics (membrane permeabilisation/pore formation, LPS binding, modulation of metabolic stress responses), making them attractive therapeutic candidates against MDR *K. pneumoniae* (especially CRKP/hvKP) infections (de Oliveira Júnior and Franco, 2020). Recent studies indicate that AMPs may be particularly valuable in biofilm-associated phenotypes; when used alone or in combination with antibiotics, they can suppress the inoculum effect and heterodirection (Lou et al., 2018; de Oliveira Júnior and Franco, 2020).

The experimental/preclinical evidence supports this potential: rationally designed, self-assembling peptides with a cationic backbone + hydrophobic core architecture have demonstrated significant bactericidal activity against *K. pneumoniae* isolates under both planktonic and biofilm conditions. It has been reported that this approach improves resistance to enzymatic degradation and the balance of toxicity/selectivity (Mwangi et al., 2025). In a comparative study, the effects of different antimicrobial peptides on *K. pneumoniae* biofilm formation, metabolic activity, and viability were systematically evaluated, and it was found that some peptides provided a distinct advantage in biofilm eradication (Hanstein et al., 2025). Due to their broad membrane-disruptive activity, several next-generation antimicrobial peptides have demonstrated more targeted mechanisms against MDR *K. pneumoniae* (Xing et al., 2025a). Peptides such as thanatin have shown the ability to interfere with NDM-mediated resistance, whereas proline-rich peptides and engineered peptide derivatives have exhibited promising activity by targeting intracellular bacterial processes while maintaining efficacy against resistant isolates. These findings highlight the increasing diversity of AMP mechanisms and support their potential as precision antimicrobial candidates (Ding et al., 2025; Xing et al., 2025a). Additionally, new candidates such as Cec4 have demonstrated therapeutic potential against resistant strains, including CRKP, by exhibiting rapid bactericidal activity in *in vitro* and *in vivo* models and biofilm inhibition at low concentrations (Li et al., 2025d). Nano-carriers and self-assembled designs have shown promise for translating AMP activity into clinical practice by increasing the bioavailability of peptides, reducing proteolytic degradation, and improving delivery to target tissues such as the lungs (Ma et al., 2024). Recent advances in nanotechnology have further enhanced the therapeutic potential of AMPs by improving peptide stability, prolonging circulation time, and facilitating targeted delivery (van der Weide et al., 2020; Suresh et al., 2023). Polymeric nanoparticles, lipid-based nano-carriers, and other advanced delivery systems have demonstrated encouraging results in experimental models by increasing antimicrobial efficacy while reducing systemic toxicity, supporting their potential for future clinical translation (van der Weide et al., 2020; Macêdo et al., 2024).

There are still barriers to clinical integration: susceptibility to proteolytic degradation, binding to serum proteins, potential cytotoxicity, and production costs are common barriers (Lou et al., 2018; de Oliveira Júnior and Franco, 2020). Several strategies have been proposed to overcome these limitations, including cyclised peptides, incorporation of D-amino acids, lipidated/derivatised peptide analogs, and nano-carrier systems (Mwangi et al., 2025). Although clinical data are limited, rational design, smart carrier technologies, and combination strategies with antibiotics suggest a trend toward the selective integration of AMPs into personalized treatment, particularly for biofilm-associated infections (Mwangi et al., 2025; de Oliveira Júnior and Franco, 2020). Emerging evidence also suggests that AMP-based combination strategies may further improve treatment outcomes. In particular, the complementary use of antimicrobial peptides with bacteriophages has attracted growing interest because disruption of the biofilm matrix and bacterial membrane by AMPs may facilitate phage penetration, while phage-derived lytic enzymes can further enhance bacterial eradication (Ding et al., 2025; Tyagi et al., 2024). Although this strategy remains largely preclinical, it represents a promising direction for the development of next-generation combination therapies against MDR *K. pneumoniae* infections (Mirski et al., 2019; Hagh Ranjbar et al., 2023).

#### 6.2.4 Antimicrobial nanoparticle technology

Antimicrobial nanoparticle technology has emerged as a promising strategy for overcoming MDR *K. pneumoniae* infections by enhancing antibacterial activity through mechanisms that differ from those of conventional antibiotics (Barani et al., 2022; Mazumder et al., 2026). Metallic and metal oxide nanoparticles, including silver (Ag), copper oxide (CuO), zinc oxide (ZnO), and gold (Au) nanoparticles, exert their antimicrobial effects by disrupting bacterial membranes, inducing oxidative stress through reactive oxygen species (ROS) generation, and damaging essential cellular proteins and nucleic acids. These multimodal mechanisms reduce the likelihood of resistance development while enabling synergistic interactions with existing antibiotics (Alotaibi et al., 2022; Arshad et al., 2025).

Recent studies have demonstrated that nanoparticle–antibiotic combinations can substantially improve therapeutic efficacy. In particular, CuO nanoparticles combined with cefepime and Ag nanoparticles used together with conventional antibiotics have shown enhanced antibacterial activity against MDR *K. pneumoniae*, accompanied by improved bacterial clearance and inhibition of biofilm formation (van der Weide et al., 2020; Alotaibi et al., 2022; Arshad et al., 2025). Likewise, advanced nano-carrier systems, including polymeric nanoparticles and lipid-based formulations, have been developed to optimize drug delivery, increase local drug concentrations, reduce systemic toxicity, and improve penetration into biofilm-associated infections (van der Weide et al., 2020). Although issues related to long-term biosafety, pharmacokinetics, and large-scale clinical translation remain to be addressed, antimicrobial nanotechnology represents one of the most promising adjunctive strategies for improving the treatment of resistant *K. pneumoniae* infections (Barani et al., 2022).

#### 6.2.5 Antisense oligonucleotides

Antisense oligonucleotides (ASOs) represent a highly targeted therapeutic approach designed to suppress bacterial gene expression by selectively binding complementary mRNA sequences. Unlike conventional antibiotics, which generally target bacterial structures or metabolic pathways, ASOs directly interfere with the expression of resistance-associated genes, thereby restoring susceptibility to existing antimicrobial agents (Xing et al., 2025a). Synthetic platforms such as peptide nucleic acids (PNAs) and phosphorodiamidate morpholino oligomers (PMOs) have shown particular promise because of their high specificity, stability, and resistance to enzymatic degradation (Xing et al., 2025a). Despite these advantages, the efficient intracellular delivery of oligonucleotides remains one of the major challenges limiting their clinical application. To overcome this barrier, several delivery platforms—including cell-penetrating peptides (CPPs), lipid-based carriers, and polymeric nano-carriers—have been developed to facilitate bacterial uptake and improve intracellular bioavailability (Abushahba et al., 2016). As advances in delivery technologies continue, ASO-based therapeutics are increasingly being recognized as a promising precision medicine strategy capable of complementing conventional antibiotics in the management of MDR *K. pneumoniae* infections (Wu et al., 2019).

#### 6.2.6 Gene editing strategies

Gene editing technologies, particularly CRISPR/Cas systems, have opened new possibilities for combating AMR by directly targeting bacterial resistance determinants. Unlike conventional antimicrobial agents, CRISPR-based approaches enable programmable inactivation of specific resistance genes, including *bla*_KPC_, *bla*_NDM_, and *bla*_OXA − 48_, or elimination of resistance plasmids, thereby restoring bacterial susceptibility to existing antibiotics (Ding et al., 2025; Baba et al., 2026). Recent studies have demonstrated that CRISPR-mediated disruption of carbapenemase genes can successfully resensitise resistant *K. pneumoniae* isolates to carbapenems both *in vitro* and *in vivo* (Ding et al., 2025; Baba et al., 2026).

In addition to the widely used CRISPR/Cas9 platform, emerging systems such as CRISPR interference (CRISPRi) and CRISPR/Cas3 provide alternative strategies for transcriptional silencing or large-scale degradation of resistance-associated genetic elements without necessarily introducing double-strand DNA breaks (Hajizadeh and Oloomi, 2025). Nevertheless, several challenges—including efficient delivery into bacterial cells, potential off-target effects, and optimisation of carrier systems—must be addressed before routine clinical application becomes feasible. Despite these limitations, CRISPR-based therapeutics are widely regarded as one of the most innovative precision antimicrobial strategies currently under development and hold considerable promise for the future management of MDR *K. pneumoniae* infections (Muteeb et al., 2025; Ding et al., 2025).

### 6.3 Role of antimicrobial stewardship

Antimicrobial stewardship (AMS) programmes have emerged as one of the most critical approaches in combating MDR pathogens such as *K. pneumoniae*, alongside the increasing prevalence of antibiotic resistance (Vijayakumar et al., 2024). The main goal of these programmes is to increase treatment effectiveness and slow down the development of resistance by ensuring that antibiotics are used correctly in terms of the right agent, the right dose, the right duration, and the right route (Carrara et al., 2021). The World Health Organization (2015) and ECDC (2024) (European Antimicrobial Resistance Surveillance Network) emphasize that AMS is a key component in reducing the burden of CRKP infections on healthcare systems, alongside infection control measures, surveillance programmes, and new drug development efforts. Antimicrobial stewardship is also increasingly being implemented within the broader One Health framework, recognizing the interconnected roles of human, animal, and environmental health in the emergence and dissemination of AMR (Chawla et al., 2025). Coordinated surveillance systems, including the WHO Global Antimicrobial Resistance and Use Surveillance System (GLASS), facilitate standardized monitoring of resistance trends and antimicrobial consumption, thereby supporting evidence-based stewardship policies at both national and international levels (Muteeb et al., 2025; Alishvandi et al., 2025).

Clinical studies have demonstrated the multifaceted benefits of AMS programmes. Tailoring empirical treatment protocols to local resistance patterns, closely monitoring antibiotic consumption, and implementing de-escalation/restriction strategies when necessary can reduce mortality rates in resistant Gram-negative infections and improve treatment success (Baur et al., 2017; Akbari et al., 2025). Restrictions on carbapenem use in some centers and the selective use of new-generation BL/BLI agents have contributed to a significant decline in CRKP rates (López-Viñau et al., 2024). Conventional culture-based susceptibility testing often requires 48–72 h, delaying appropriate therapy and frequently leading to unnecessary exposure to broad-spectrum antibiotics. Therefore, integrating rapid diagnostic technologies into AMS—including multiplex PCR assays, MALDI-TOF mass spectrometry, and other molecular platforms capable of rapidly identifying major carbapenemase genes—enables earlier initiation of targeted antimicrobial therapy, improves clinical outcomes, and reduces unnecessary antibiotic consumption and healthcare costs (Banerjee and Humphries, 2017).

There are several challenges to the widespread adoption of AMS. In particular, resource and training shortages, limited laboratory capacity in developing countries, and difficulties in changing prescribing habits make it challenging to sustain programmes (Abbas, 2024). Prominent strategies for the future include artificial intelligence-supported decision algorithms, microbiome-based treatment plans, and personalized antibiotic management using pharmacogenetic data. The integration of electronic health records with real-time antimicrobial consumption and resistance surveillance is expected to further strengthen stewardship programmes by supporting timely clinical decision-making (Muteeb et al., 2025). In parallel, advances in artificial intelligence and big-data analytics may enable the development of predictive models capable of integrating patient characteristics, local resistance epidemiology, and pharmacological data to optimize individualized antimicrobial selection and dosing (Xing et al., 2025a; Alishvandi et al., 2025). These developments have the potential to transform AMS from merely a tool that slows down resistance development into an innovative and dynamic treatment platform for combating resistant *K. pneumoniae* (Abbas, 2024; Pennisi et al., 2025).

## 7 Prevention and control strategies

### 7.1 Infection control measures in hospitals

*K. pneumoniae* is a natural constituent of the human intestinal microbiota, yet it represents a major contributor to HAIs. These infections often prolong hospital stays due to therapeutic challenges and substantially increase healthcare costs (Martin and Bachman, 2018; Silvotti et al., 2025; Martin et al., 2016). *K. pneumoniae* readily acquires AMR through diverse mechanisms. Based on virulence factors and resistance profiles, strains are categorized as CR-hvKP or cKP (Martin and Bachman, 2018). Carbapenemase-producing *K. pneumoniae* has become endemic in various regions, including Europe (Poland, Greece, Italy, and Spain), Latin America, North America, South America, and Asia (Falagas et al., 2025). Developing effective infection prevention and control strategies is essential to mitigate the high morbidity and mortality associated with *K. pneumoniae* infections globally (Lee et al., 2016; Vock and Tschudin-Sutter, 2019). As an opportunistic pathogen, *K. pneumoniae* predominantly affects hospitalized patients or individuals with compromised immune systems. It accounts for approximately 83% of ventilator-associated infections and contributes to 20%−30% of deaths related to bloodstream infections (Gorrie et al., 2022).

Sources of transmission of *K. pneumoniae* infections in hospitals include the hands of healthcare workers, person-to-person contact between patients, and contamination of used equipment and surfaces (Martin and Bachman, 2018). *K. pneumoniae* colonization plays a role in the development of infection. Studies have shown that 5% of colonized patients develop infections, and there is an 80% concordance between the colonized strain and the hospital-acquired strain (Martin et al., 2016; Gorrie et al., 2022). These results show that colonization is a step in the development of infection; therefore, identifying colonized patients and taking the necessary precautions is of great importance in preventing the development of infection. HAIs are infections that occur at least 48 h after hospital admission (Martin and Bachman, 2018). To prevent these infections, it is crucial to train hospital staff (especially those in ICUs) on cleanliness and hygiene, and to ensure that the hospital environment and equipment used are cleaned correctly and adequately. Cleaning personnel should wear clean aprons and maintain good hand hygiene before preparing beds or distributing cleaning supplies. Care should be taken to use personal protective equipment such as aprons, gloves, and masks properly (Verma and Sharma, 2025). To prevent the spread of resistant strains in hospitals, it is crucial to rapidly identify the resistance profiles of these strains using techniques such as PCR or Whole Genome Sequencing (WGS), implement appropriate treatment protocols, and prevent contact between these patients and others for infection control (Silvotti et al., 2025). Uncontrolled and widespread use of β-lactam antibiotics, in particular, leads to the emergence of resistant strains, which increases mortality and morbidity. Identifying appropriate and necessary treatment methods and implementing treatment protocols for infection control is crucial (Verma and Sharma, 2025; Oliveira et al., 2024) (Table 4).

**Table 4:** Prevention and control strategies for *Klebsiella pneumoniae* infections and antimicrobial resistance.

Category	Specific strategy/ application area	Primary objective	Priority target clinical/ epidemiological	References
Infection prevention and hospital control	Hand hygiene, PPE use, surface and device decontamination, staff training in ICUs, early identification of colonized patients	Prevent hospital transmission and reduce HAIs caused by MDR/CRKP	ICU patients, patients with catheters/ventilators, high-prevalence hospital units	Martin and Bachman, 2018; Silvotti et al., 2025; Martin et al., 2016; Gorrie et al., 2022; Verma and Sharma, 2025
Early detection and rapid response	Rapid molecular diagnostics (PCR, WGS), screening for carbapenemase producers, patient isolation/cohorting	Early containment of resistant strains and faster optimisation of therapies	Known CRKP carriers, outbreak clusters, high-risk admissions	Silvotti et al., 2025; Martin et al., 2016; Verma and Sharma, 2025; Oliveira et al., 2024
Antimicrobial stewardship programmes	Antibiotic optimisation, de-escalation, reducing unnecessary β-lactam/carbapenem use; integration of rapid diagnostics	Reduce selective pressure and prevent emergence/spread of MDR/CRKP	Hospitalized patients receiving broad-spectrum antibiotics; units with high carbapenem use	Carrara et al., 2021; World Health Organization, 2015; ECDC, 2024; Baur et al., 2017; Akbari et al., 2025; López-Viñau et al., 2024; Banerjee and Humphries, 2017; Abbas, 2024; Pennisi et al., 2025
Surveillance and monitoring systems	Participation in GLASS, EARS-Net, CDC AR Lab Network; tracking high-risk clones (ST258, ST147, ST307, ST512)	Detect evolving resistance patterns and guide empirical therapy	National/regional health systems; hospitals during outbreak risk	Wyres and Holt, 2018; Cantón et al., 2023; ECDC, 2024; Oliveira et al., 2024; World Health Organization, 2022b; Lessa and Sievert, 2023; Jean et al., 2022; World Health Organization, 2018; Xanthopoulou et al., 2022; European Centre for Disease Prevention and Control (ECDC), 2024; Ajulo and Awosile, 2024
One Health approach	Integrated surveillance across humans, animals, food, environment; GLASS-EAR program	Prevent cross-sectoral AMR transmission and monitor environmental reservoirs	Livestock systems, wastewater, companion animals, agriculture-environment interface	Jesudason, 2024; Bertagnolio et al., 2024; World Health Organization (WHO), 2025; Zhang J. et al., 2025; Aslam et al., 2021; World Health Organization et al., 2022
Global policy and international collaboration	WHO Global Action Plan on AMR; global pathogen priority list; strengthening laboratory capacity	Coordinate global preparedness, improve diagnostics, and support AMR research investment	Countries with high CRKP burden; LMICs with limited AMR infrastructure	Jesudason, 2024; Bertagnolio et al., 2024; World Health Organization (WHO), 2025; World Health Organization et al., 2022
Rational antibiotic use and restriction policies	Reducing inappropriate β-lactam, aminoglycoside, and fluoroquinolone use	Minimize selective pressure driving MDR and CRKP emergence	High-prescribing clinical units; patients receiving repeated antibiotic courses	Agyeman et al., 2020; Van Giang et al., 2025; Bai et al., 2022; Durdu et al., 2019; Arato et al., 2021; Oliveira et al., 2024
Outbreak prevention and control	MLST typing for early outbreak detection, cohort isolation, reinforcement of contact precautions	Rapid interruption of clonal spread	Units experiencing sudden increases in CRKP; long-term care hospitals	Wyres and Holt, 2018; Rizvi et al., 2025; David et al., 2019
Environmental cleaning and device disinfection	Routine high-level disinfection of equipment and surfaces; proper cleaning protocols	Minimize environmental reservoirs of MDR strains	ICUs, wards with high device use (catheters, ventilators)	Verma and Sharma, 2025
Protecting high-risk clinical populations	Screening, minimizing invasive procedures, enhanced monitoring	Reduce morbidity and mortality in vulnerable groups	Elderly, neonates, immunocompromised, ICU patients	Jung et al., 2012; Juan et al., 2019; Peirano et al., 2020; Gorrie et al., 2017; Wang X. et al., 2025
Precision medicine and biofilm-aware treatment	Considering biofilm phenotype, virulence factors, and resistance markers in therapy selection	Improve treatment success in biofilm-associated and severe infections	Chronic infections, device-associated infections, recurrent infections	Karampatakis et al., 2023; Mohammed et al., 2023; Elbehiry et al., 2025
Research, innovation and future therapeutics	Development of vaccines, bacteriophages, AMPs, nanoparticles; new antibiotics	Provide future alternatives for CRKP and MDR *K. pneumoniae*	Global CRKP hotspots; regions with high carbapenemase prevalence	Xing et al., 2025b; Mwangi et al., 2025; Yu Y. et al., 2025; Li L. et al., 2024; Xing et al., 2025a; Yu J. et al., 2025; Lu et al., 2025; de Oliveira Júnior and Franco, 2020; Hanstein et al., 2025; Li et al., 2025d; Ma et al., 2024; World Health Organization (WHO), 2025

PPE, personal protective equipment; ICU, intensive care units; HAI, healthcare-associated infections; MDR, Multi-Drug Resistant; CRKP, carbapenem-resistant K. pneumoniae; PCR, polymerase chain reaction; WGS, whole genome sequencing; GLASS, Global Antimicrobial Resistance Surveillance System; EARS-Net, European Antimicrobial Resistance Surveillance Network; CDC AR Lab Network, Centers for Disease Control and Prevention Antibiotic Resistance Laboratory Network; GLASS-EAR, GLASS-Emerging Antimicrobial Resistance Reporting System; WHO, World Health Organization; LMIC, Low- and Middle-Income Countries; MLST, multilocus sequence typing; AMR, antimicrobial resistance; AMP, antimicrobial peptides.

### 7.2 Surveillance and monitoring programs

*K. pneumoniae* is a major opportunistic pathogen associated with diverse infections, particularly in hospital environments. AMR in this species is primarily driven by the acquisition of β-lactamase genes, including ESBLs such as *bla*_CTX − M_, *bla*_SHV_, and *bla*_TEM_, as well as carbapenemases like *bla*_KPC_, *bla*_NDM_, and *bla*_OXA − 48_. These resistance determinants are frequently plasmid-borne, promoting horizontal gene transfer across strains and species (Martin and Bachman, 2018). The widespread dissemination of high-risk epidemic clones, notably ST258, ST147, and ST512, has further intensified the clinical burden (Wyres and Holt, 2018). The global rise of MDR, XDR, and CRKP represents a critical threat to public health. In recent decades, the rise of antimicrobial resistance, particularly carbapenem resistance, has elevated this organism to a critical priority pathogen (Cantón et al., 2023; Mancuso et al., 2023). Surveillance and monitoring programs are essential for understanding the epidemiology of resistant strains, guiding antibiotic stewardship, and informing infection control strategies (Oliveira et al., 2024). Surveillance and monitoring systems are essential pillars in the global fight against AMR, particularly in pathogens such as *K. pneumoniae*, which have demonstrated a high capacity for acquiring and disseminating resistance genes. Effective surveillance allows for the timely identification of emerging resistance patterns, facilitates outbreak detection, and guides both empirical treatment and long-term public health interventions (Cantón et al., 2023; Falagas et al., 2025). One of the primary objectives of surveillance is to identify AMR trends early. Continuous monitoring of *K. pneumoniae* isolates obtained from clinical specimens allows for the timely detection of rising resistance rates to critical antibiotics, including carbapenems and third-generation cephalosporins (Rizvi et al., 2025). Such surveillance is essential for preventing the dissemination and establishment of resistant clones within healthcare settings. Without surveillance, emerging threats such as OXA-48- or NDM-producing strains may remain undetected until they become widespread and more difficult to control (David et al., 2019).

In many healthcare settings, empirical antibiotic treatment is initiated before definitive culture and susceptibility results are available. Local and regional surveillance data on *K. pneumoniae* resistance profiles provide clinicians with the necessary information to select the most appropriate empirical therapy. This reduces the use of broad-spectrum antibiotics and improves treatment outcomes, especially in critical care settings where time-sensitive decisions are required (Martin and Bachman, 2018). Surveillance systems, especially those incorporating molecular typing tools, are critical for identifying outbreaks caused by the clonal expansion of high-risk *K. pneumoniae* lineages (e.g., ST258, ST307). Early recognition of outbreaks allows infection control teams to implement measures such as cohorting, contact precautions, and environmental cleaning to prevent further transmission. Delayed recognition can lead to hospital-wide or even interhospital spread (Wyres and Holt, 2018). Surveillance data serve as a valuable tool for evaluating the effectiveness of infection prevention and control (IPC) interventions. For example, a decline in the incidence of CRKP observed after the introduction of hand hygiene initiatives or AMS programmes provides direct evidence of the success of such measures (Vock and Tschudin-Sutter, 2019).

Continuous monitoring ensures that interventions remain effective and responsive to evolving epidemiology [Vock and Tschudin-Sutter, 2019; Centers for Disease Control Prevention (CDC), 2023]. On a broader scale, surveillance data are essential for informing national and international public health strategies. They serve as the evidence base for AMR action plans, funding allocation, vaccine development, and prioritization of research efforts. Organizations such as the WHO, ECDC, and CDC rely on surveillance data to determine which bacterial pathogens pose the greatest threat globally and how to respond to them (Prestinaci et al., 2015). In an interconnected world, resistant *K. pneumoniae* strains can rapidly spread across borders via patient transfers, travel, or trade. Surveillance fosters international collaboration through standardized data collection and timely sharing. Systems such as WHO Global Antimicrobial Resistance Surveillance System (GLASS) and ECDC's European Antimicrobial Resistance Surveillance Network (EARS-Net) enable global comparisons, facilitating the identification of cross-border threats and the coordination of responses. *K. pneumoniae* has been increasingly detected in animal, environmental, and food sources, raising concerns about zoonotic and ecological reservoirs of resistance. Integrating *K. pneumoniae* surveillance into One Health frameworks allows for a more comprehensive understanding of resistance ecology. This interdisciplinary approach enhances the ability to intervene at multiple levels: human, animal, and environmental (Oliveira et al., 2024; World Health Organization, 2023) (Table 4).

#### 7.2.1 Global surveillance programs

Global surveillance systems are crucial for tracking the epidemiology of antimicrobial-resistant *K. pneumoniae* and for facilitating coordinated international responses. These programs aim to harmonize data collection across regions, detect emerging threats, and guide clinical and public health decision-making. The most significant global initiatives include the WHO's GLASS, the ECDC's EARS-Net, the CDC's Antibiotic Resistance Laboratory Network (AR Lab Network), and region-specific surveillance efforts in Asia, Africa, and Latin America (World Health Organization, 2023; World Health Organization et al., 2022; Lessa and Sievert, 2023; Jean et al., 2022).

##### 7.2.1.1 GLASS

The WHO launched GLASS in 2015 as the first standardized global system for collecting and analyzing AMR data across countries. GLASS promotes uniformity in surveillance by establishing guidelines for case definitions, specimen types (e.g., blood, urine), priority pathogens and antibiotics, laboratory methods, and reporting standards (World Health Organization, 2018). *K. pneumoniae* is among the eight priority bacterial pathogens monitored by GLASS. The system focuses on resistance to third-generation cephalosporins, carbapenems, and fluoroquinolones (World Health Organization, 2022b). As of 2023, over 100 countries have enrolled in GLASS, although the completeness and consistency of data still vary. GLASS data have shown alarming global trends in *K. pneumoniae* resistance. In some countries, over 50% of isolates are resistant to third-generation cephalosporins. Carbapenem resistance exceeds 25% in certain high-burden regions (Prestinaci et al., 2015; Xanthopoulou et al., 2022). GLASS also supports capacity-building through training, quality assurance programs, and the development of national surveillance frameworks (World Health Organization, 2018).

##### 7.2.1.2 EARS-Net

The EARS-Net, coordinated by the ECDC, represents the largest publicly funded AMR surveillance initiative in Europe (Oliveira et al., 2024). The network primarily monitors invasive bacterial isolates, particularly from blood and cerebrospinal fluid, across more than 30 participating countries. *K. pneumoniae* is one of the core pathogens tracked. EARS-Net collects data on susceptibility to key antibiotics such as cefotaxime, ceftriaxone (3rd-gen cephalosporins), imipenem, meropenem (carbapenems), ciprofloxacin, and aminoglycosides (gentamicin, amikacin). According to the latest 2023 report, carbapenem resistance in *K. pneumoniae* continues to rise, particularly in southern and eastern European countries such as Greece, Italy, and Romania. Additionally, MDR strains are now common in hospital settings (Xanthopoulou et al., 2022; European Centre for Disease Prevention and Control (ECDC), 2024). EARS-Net data inform AMR control and contribute to joint actions on antimicrobial stewardship and infection prevention (Xanthopoulou et al., 2022).

##### 7.2.1.3 Antibiotic resistance laboratory network

In the United States, the CDC established the Antibiotic Resistance Laboratory Network (AR Lab Network) to enable the rapid detection, monitoring, and containment of antibiotic-resistant threats, including CRKP. The AR Lab Network provides real-time identification of resistance genes (e.g., *bla*_KPC_, *bla*_NDM_, *bla*_OXA − 48_), supports local outbreak investigations, performs WGS of resistant strains, and facilitates the tracking of interfacility transmission. The CDC considers carbapenem-resistant Enterobacterales (CRE), including CRKP, to be a significant and urgent threat. According to 2023 data, CRE causes approximately 13,000 infections annually in the US Mortality rates can exceed 40% in bloodstream infections (Centers for Disease Control Prevention (CDC), 2023; Caliskan-Aydogan and Alocilja, 2023). The CDC also runs the National Antimicrobial Resistance Monitoring System (NARMS), which monitors AMR in foodborne pathogens and shares early warning signals of emerging resistance that may impact *K. pneumoniae* indirectly through environmental or zoonotic pathways (Caliskan-Aydogan and Alocilja, 2023).

##### 7.2.1.4 Regional programs in Asia, Africa, and Latin America

Beyond the global platforms, several regional networks provide valuable insights into *K. pneumoniae* resistance. The Asian Network for Surveillance of Resistant Pathogens (ANSORP) and the WHO's South-East Asia AMR Network track the rising rates of ESBL- and carbapenemase-producing strains in countries such as India, China, and Vietnam (Navon-Venezia et al., 2017). Surveillance is often limited by infrastructure, but initiatives such as the Africa CDC AMR Surveillance Program and WHO AFRO regional networks are working to build capacity. Studies from North Africa report high rates of OXA-48-producing *K. pneumoniae* (Caliskan-Aydogan and Alocilja, 2023). The ReLAVRA network (coordinated by PAHO/WHO) supports laboratory-based surveillance in over 20 countries. Widespread KPC-producing *K. pneumoniae* strains have been documented, particularly in Brazil, Argentina, and Colombia (Navon-Venezia et al., 2017; Falagas et al., 2025; World Health Organization, 2020).

#### 7.2.2 The role of molecular surveillance

Modern surveillance systems increasingly integrate molecular methods, such as WGS, PCR-based resistance gene detection, plasmid and transposon mapping, and multilocus sequence typing (MLST). Molecular surveillance enables real-time outbreak tracking, monitoring of clonal lineages (e.g., ST258, ST147), and a deeper understanding of horizontal gene transfer dynamics. Countries with advanced WGS platforms, such as the UK (via the COG-UK initiative), have successfully used genomics to contain the nosocomial spread of resistant *K. pneumoniae* strains (David et al., 2019).

### 7.3 Vaccine development prospects

*K. pneumoniae* is the fourth leading cause of death from infections, responsible for 790,000 fatalities across all age groups. *K. pneumoniae* is the second leading cause of death due to AMR worldwide and the first in Sub-Saharan Africa (Murray et al., 2022; Dangor et al., 2024). Neonates, young infants, and the elderly are among the most at-risk groups for *K. pneumoniae* infections. Individuals with underlying medical conditions, immunocompromised individuals, those receiving intravascular devices or mechanical ventilator support, and those in ICUs are also at risk. The *K. pneumoniae* vaccine could prevent 4 million deaths annually due to AMR. Furthermore, AMR-based treatment could reduce the duration of hospital stays and the cost of intensive care unit admissions (Dangor et al., 2024).

Several biological and epidemiological factors complicate the development of an effective vaccine against *K. pneumoniae*. The bacterium exhibits a high degree of genetic and antigenic diversity, particularly in its capsular polysaccharide (CPS) and LPS structures (World Health Organization, 2020). Over 80 distinct capsular types (K) and multiple O-antigen types have been identified, making broad-spectrum vaccine design difficult. Additionally, the coexistence of hypervirulent and MDR strains adds another layer of complexity, as vaccines must ideally confer protection against both types (Wang Q. et al., 2025). The identification of suitable antigenic targets is one of the most critical steps in developing an effective vaccine against *K. pneumoniae*. Given the bacterium's complex virulence arsenal and its high level of genomic diversity, antigen selection must strike a balance between broad strain coverage, immunogenicity, and safety. Several key antigen classes have been explored as potential vaccine components, each with distinct advantages and limitations (Hetta et al., 2025). Efforts have focused primarily on surface antigens that play roles in immune evasion and pathogenesis. The most promising targets include CPS, which are major virulence factors that elicit protective immunity (Opoku-Temeng et al., 2019). Among these, multivalent vaccines targeting the most prevalent K-antigen types (e.g., K1, K2, K5, K20) have shown promise in preclinical studies (Russo and Marr, 2019). LPS has received less attention in *K. pneumoniae* vaccine development than CPS. This is due to uncertainty about whether these polysaccharides will remain in contact with the surface in the presence of the capsule and whether protective antibodies will recognize them. However, studies by two independent groups have resulted in the development of specific anti-O polysaccharide (OPS) sera against *K. pneumoniae* isolates. Nine unique O-antigen serogroups have emerged. The majority (79%) of clinical infections were caused by O1, O2, O3, and O5 types. This demonstrated that a quadrivalent OPS-based vaccine is more commercially viable than a 24-valent CPS-based vaccine (Miller et al., 2024).

O-Antigens of LPS have the advantage of broader coverage, as fewer O-types dominate clinical isolates globally. OMPs are crucial components of the bacterial outer membrane, playing a vital role in nutrient uptake, maintaining structural integrity, and facilitating interaction with the host environment. Some of these proteins are highly conserved across strains and are immunogenic, making them attractive targets for vaccine development (Opoku-Temeng et al., 2019; O'Donoghue et al., 2017). Prominent OMP candidates include OmpA, OmpK35, OmpK36, OmpK17, and OmpK37. OmpA is a porin involved in host cell adhesion and the formation of biofilms. It is widely conserved and elicits strong antibody responses. OmpK35 and OmpK36 are channels involved in antibiotic permeation and may contribute to resistance mechanisms. OmpK17 and OmpK37 play a crucial role in iron acquisition, a vital factor for bacterial growth within the host. Recombinant OMP or peptide-based vaccines have shown partial protection in animal models and may be particularly useful when combined with polysaccharide antigens (Goodarzi et al., 2021; Opoku-Temeng et al., 2019; Rocker et al., 2020). Fimbriae, or pili, are filamentous surface structures that mediate bacterial adhesion to host cells and abiotic surfaces, playing a pivotal role in colonization and biofilm formation. *K. pneumoniae* expresses at least two well-characterized fimbrial types (Xu et al., 2024; Isidro-Coxca et al., 2024).

Type 1 fimbriae (encoded by the fim operon) are involved in urinary tract infections by promoting adhesion to urothelial cells. Type 3 fimbriae (encoded by the mrk operon) facilitate biofilm formation on medical devices and epithelial surfaces, contributing to chronic infections and resistance (Xu et al., 2024; Assoni et al., 2025). Adhesin subunits such as FimH and MrkD have been explored as subunit vaccine antigens. These proteins are surface-exposed and relatively conserved, and antibodies directed against them can block bacterial attachment, offering a non-bactericidal but protective mechanism (Assoni et al., 2025). Siderophore receptors located on the bacterial outer membrane are accessible to the host immune system and represent attractive targets for vaccine development. For instance, IroN (salmochelin receptor), IutA (aerobactin receptor), and FyuA (yersiniabactin receptor) have been shown to be immunogenic and protective in murine infection models. Targeting iron uptake systems has the added advantage of impairing bacterial growth in the host without exerting selective pressure that typically drives resistance (Hetta et al., 2025; Wang J. et al., 2020; Gorden et al., 2018).

Polysaccharide-protein conjugate vaccines, which combine CPS or O-antigens with carrier proteins (e.g., CRM197 or tetanus toxoid) to trigger T-cell-dependent immune responses, are among the vaccine candidates (Wantuch et al., 2023). Some candidates are in early-phase clinical trials. Outer membrane vesicle-based vaccines contain a wide variety of surface antigens and have demonstrated immunogenicity in animal models (Li et al., 2025c). State-of-the-art platforms, such as mRNA and DNA vaccines, are still in the early stages of research for *K. pneumoniae* but offer the potential for rapid design and production. Live attenuated and inactivated vaccines, while potentially effective, are limited by safety concerns related to virulence reversion or adverse immune responses (Wang Q. et al., 2025).

The development of a safe and effective vaccine against *K. pneumoniae* represents a crucial component in the global fight against antimicrobial resistance. While significant challenges remain, recent advances in molecular biology, immunology, and vaccine technology offer promising tools to overcome these hurdles (Douradinha, 2024). A successful vaccine would not only prevent severe infections but also reduce reliance on antibiotics, helping to curb the rise of resistant strains. Continued interdisciplinary collaboration and investment are vital to realizing the potential of *K. pneumoniae* vaccination (Hetta et al., 2025; Wang Q. et al., 2025).

## 8 Future perspectives and research directions

### 8.1 Advances in genomic and molecular research

Due to the widespread prevalence of MDR *K. pneumoniae* strains and the limited availability of effective therapeutic options, it is essential to determine both the phenotypic and genotypic resistance profiles of clinical isolates. This not only aids in developing preventive strategies but also facilitates the identification of resistance mechanisms, detection of clonal spread, and tracing of infection sources to control outbreaks (Snitkin et al., 2012).

Conventional genotyping techniques, such as pulsed-field gel electrophoresis (PFGE) and MLST, are commonly employed to investigate whether widespread infections represent outbreaks. While these methods provide sufficient discriminatory power for cKp strains, they are less effective in distinguishing highly clonal strains. WGS has therefore emerged as the gold standard for bacterial typing, offering superior resolution. WGS allows for the simultaneous determination of species, sequence type, O- and K-loci, AMR determinants, and virulence factors, thereby providing a comprehensive picture of *K. pneumoniae* epidemiology. Despite the genetic diversity and complexity of this pathogen, WGS has enabled the identification and monitoring of emerging epidemic clones (Snitkin et al., 2012; Wyres et al., 2020; Mejía-Limones et al., 2024).

In addition, transcriptomic approaches, which analyse global gene expression under specific environmental conditions (e.g., antibiotic exposure), provide insights into the molecular basis of resistance. Recent studies have reported upregulation of genes linked to cell wall biosynthesis and LPS production, alongside activation of metabolic pathways such as butanoate metabolism, in resistant *K. pneumoniae* strains (Liu et al., 2025; Dai et al., 2025). These findings suggest that AMR arises not only from the horizontal acquisition of resistance genes but also from the regulation of intrinsic metabolic and stress-response pathways (Liu et al., 2025).

Proteomic analyses further expand this understanding by profiling the bacterium's full protein complement, including virulence-associated proteins and host–pathogen interaction factors (Illenseher et al., 2025; Nenni et al., 2020). Although the thick capsular layer of *K. pneumoniae* shields OMPs from immune recognition, proteomic investigations have identified several OMPs, including Pal and SlyB, as critical for virulence and bacterial fitness. These proteins are also considered promising immunogenic targets and potential vaccine candidates (Illenseher et al., 2025) (Table 4).

### 8.2 Precision medicine and personalized therapy

*K. pneumoniae* can readily acquire and transmit AMR genes using horizontal gene transfer methods such as conjugation, transformation, transduction, and transposition. These characteristics are associated with increased mortality and morbidity in *K. pneumoniae* infections (Mohammed et al., 2023). In particular, when patients with underlying conditions or immunosuppression become infected with resistant *K. pneumoniae*, treatment becomes more difficult, and mortality rates increase significantly (Karampatakis et al., 2023; Riaz et al., 2021).

The implementation of new treatment approaches, such as precision medicine and personalized therapy, is crucial for reducing mortality and morbidity rates and facilitating treatment. According to the definition of the US National Human Genome Research Institute, precision medicine and personalized therapy encompass medical methods that take into account an individual's lifestyle, environmental, and genomic information (Delpierre and Lefèvre, 2023). Precision medicine and personalized therapy aim to determine the most appropriate treatment strategy based on the genetic and molecular characteristics of each infection and each patient (Wang and Wang, 2023).

Traditional methods for determining pathogen resistance profiles before treatment require an incubation period of 16–20 h for antibiotic susceptibility testing, which is too long for critical cases. To address this issue, rapid disk diffusion tests (4–8 h) have been developed from positive blood culture bottles. Innovative phenotypic tests, such as chromogenic media, also save time, allowing for rapid patient-specific tailoring of empiric therapy (Gajic et al., 2022; Hattab et al., 2024). However, phenotypic tests have limitations, including differences in sensitivity and specificity, as well as the influence of structures such as biofilms on the results of antibiotic susceptibility tests. Genomic diagnostics have addressed these limitations (Hattab et al., 2024; Muntean et al., 2022). These tests, which directly detect specific bacterial resistance genes such as *bla*_KPC_ and *bla*_NDM_, can identify the genetic basis for resistance that phenotypic tests may miss or that emerges later. This allows for more accurate predictions of the course of infection and more reliable treatment guidance. Therefore, a hybrid diagnostic approach that combines rapid phenotypic testing with in-depth genotypic analysis is essential for making optimal treatment decisions (Elbehiry et al., 2025).

Next-Generation Sequencing (NGS) technologies, particularly WGS and metagenomic NGS (mNGS) approaches, hold the potential to revolutionize the management of *K. pneumoniae* infections (Elbehiry et al., 2025; Wan et al., 2024).

WGS analyses the entire genetic make-up of a pathogen with ultra-fast speed and high accuracy, providing rich information about resistance and virulence genes. This technology is a critical tool in guiding infection control strategies not only for individual patients but also at the hospital and public health levels (Elbehiry et al., 2025). For example, monitoring clonal spread in hospital outbreaks can identify the transmission routes and rates of spread of clones such as ST11 and ST258, allowing for effective measures to interrupt the chain of transmission (Wang et al., 2023). Metagenomic NGS, on the other hand, can quickly identify even pathogens that cannot be detected by culture by analyzing the genetic material of all microorganisms found at the source of infection. mNGS demonstrates high accuracy, especially in the diagnosis of bloodstream infections, and stands out as a powerful complementary tool that aids in diagnosis (Elbehiry et al., 2025; Wang et al., 2023).

In the treatment of *K. pneumoniae* infections, empiric treatment protocols are often determined based on regional susceptibility data. However, these general data may not fully reflect the genetic resistance mechanisms possessed by a patient's specific strain (Navon-Venezia et al., 2017). Therefore, a precision medicine approach aims to optimize treatment decisions based on genotypic information obtained through genomic analysis. Technologies such as NGS provide rich and unique details on a pathogen's entire genome (Xanthopoulou et al., 2022). These analyses can rapidly and precisely identify carbapenemase genes, such as *bla*_KPC_, *bla*_NDM_, *bla*_OXA − 48_, *bla*_VIM_, and *bla*_IMP_, in a strain isolated from a patient, thereby transforming treatment into targeted therapy much earlier than would be possible with traditional methods (Elbehiry et al., 2025). These genomic analyses can also reveal other resistance mechanisms, such as porin mutations. This information allows clinicians to avoid unnecessary antibiotic use and quickly transition resistant strains to last-resort antibiotics, such as colistin or ceftazidime-avibactam (Del Rio et al., 2023; Kaya et al., 2025). Furthermore, biofilm formation is a key factor that makes bacteria 100–1,000 times more resistant to conventional antibiotic therapy, and laboratory susceptibility results may not always predict treatment success (Karampatakis et al., 2023; Mohammed et al., 2023). This highlights the importance of a precision medicine approach that provides a holistic perspective, considering not only genetic resistance but also additional pathogenic characteristics of the pathogen, such as biofilm formation (Elbehiry et al., 2025) (Table 4).

### 8.3 Policy and global collaboration efforts

AMR represents a global public health crisis with profound clinical and economic consequences. In recognition of its growing threat, the WHO member states adopted a Global Action Plan on AMR in 2015 (Bertagnolio et al., 2024). A key milestone in this framework was the publication of the WHO priority pathogens list in 2017, which was designed to guide research and development efforts and support broader public health interventions. Given the rapid evolution of resistance mechanisms and persisting data gaps, particularly in low- and middle-income countries, the WHO released an updated list in 2022. This revision, informed by the first peer-reviewed global estimates of the AMR burden, identified 24 bacterial species across 15 families of antibiotic-resistant pathogens. These were categorized into three priority tiers: critical, high, and medium, based on their clinical impact, treatment challenges, and global distribution (Jesudason, 2024; Bertagnolio et al., 2024).

The 77th World Health Assembly (WHA) adopted a resolution (May 2024) mandating the systematic collection of national and global AMR data for the period 2025–2035. The resolution also urges WHO Member States to enhance research and innovation in infection prevention, antimicrobial stewardship, and therapeutic strategies [Bertagnolio et al., 2024; World Health Organization (WHO), 2025]. *K. pneumoniae* represents a particularly urgent challenge. Classified by the WHO as a critical threat to human health, it is the third leading cause of AMR-related mortality worldwide, directly responsible for an estimated 150,000 deaths in 2019 and contributing to more than 600,000 global deaths. Its clinical relevance is heightened by the increasing convergence of MDR *K. pneumoniae* and hvKP lineages, which drive severe community- and HAIs (Braun et al., 2024). Addressing this escalating threat requires coordinated international action. Key priorities include strengthening laboratory infrastructure in low- and middle-income countries, developing a consensus definition of hvKP (Hetta et al., 2025), and supporting innovative therapeutic approaches such as phage therapy and vaccine research (Zhang J. et al., 2025). Moreover, robust implementation of AMS programmes and IPC strategies remains essential (Hetta et al., 2025).

Given the complex drivers of AMR emergence, a One Health perspective is indispensable. This framework acknowledges the interdependence of human, animal, plant, and environmental health and underscores the need for cross-sectoral collaboration to sustain global health security (Aslam et al., 2021).

At the global level, WHO collaborates with the Food and Agriculture Organization (FAO), the World Organization for Animal Health (WOAH), and the United Nations Environment Programme (UNEP) through the Quadripartite Alliance, which coordinates multi-sectoral action against AMR (World Health Organization et al., 2022). Within this framework, surveillance initiatives such as the GLASS and the GLASS-Emerging Antimicrobial Resistance Reporting System (GLASS-EAR) have been implemented to enable rapid detection of novel resistance mechanisms and outbreaks (Ajulo and Awosile, 2024). In early 2024, GLASS-EAR launched a global data call regarding hvKP isolates carrying carbapenemase genes, which confirmed the ST23 lineage in at least one country across six WHO regions (World Health Organization, 2022a).

In Europe, the ECDC released a rapid risk assessment on carbapenem-resistant Enterobacteriaceae (February 2025). The report recommends the establishment of multidisciplinary national task forces, development of comprehensive management plans with defined targets and resources, and more vigorous enforcement of IPC and stewardship policies, which are currently insufficient in most healthcare settings (ASSESSMENT RR, 2025).

In the United States, the introduction of the Pioneering Antimicrobial Subscriptions To End Upsurging Resistance (PASTEUR) Act represents a novel legislative initiative aimed at overcoming market failures in antibiotic development. If enacted, it would allow the Department of Health and Human Services (HHS) to establish subscription-based agreements for critical antimicrobials, thereby incentivising innovation while limiting overuse (ACCESS Campaign, 2025).

In contrast to global trends, China's national AMR program demonstrates that coordinated policies can effectively curb antibiotic resistance. Data from the China Antimicrobial Surveillance Network (CHINET) reported sharp increases in CRKP between 2015 and 2019, but since 2021, a declining trend has been documented, underscoring the importance of sustained national commitment (Li et al., 2025a).

This success was made possible in 2011 when the Chinese government initiated an unprecedentedly strict protocol for the use of clinical antibiotic agents, significantly reducing antibiotic use. This policy was implemented through planned and systematic interventions, such as the Quality Control Circle (QCC) project, which aimed to improve the rational use of carbapenems using a multidisciplinary collaborative model. This multidisciplinary approach, combined with strict regulatory oversight, demonstrates the power of political will and coordinated action in reversing the trend of AMR (Li et al., 2021).

As emphasized by the ECDC, the consistent application of IPC strategies is essential to curb the dissemination of AMR. Nevertheless, their implementation remains suboptimal in the majority of healthcare facilities, revealing a persistent gap between established recommendations and clinical reality (ECDC, 2024). Socioeconomic determinants, particularly poverty, play a decisive role in driving the emergence and propagation of AMR. In many low-income settings, limited access to essential healthcare services and the high costs of treatment often compel patients to postpone professional care, rely on self-medication, or resort to substandard or inappropriate therapies. These behaviors create substantial selective pressure on bacterial populations, fostering resistance. Additionally, environmental factors, including widespread pollution and poorly regulated antibiotic use in both human and veterinary medicine, accelerate the circulation of resistance genes in low- and middle-income countries (LMICs). The absence of basic infrastructure, including safe water, sanitation, and hygiene (WASH) services, intensifies the burden of infectious diseases that require antibiotic treatment, amplifying antibiotic use and fuelling the cycle of resistance development. These structural deficiencies within LMICs represent not only a regional problem but also a direct threat to global health security, as modern patterns of human mobility enable resistant strains to disseminate rapidly across borders (Salam et al., 2023; Kapatsa et al., 2025).

This scenario highlights the urgent necessity of a One Health framework, recognizing that vulnerabilities in any single region, whether linked to human, animal, or environmental health, can swiftly escalate into a worldwide challenge (ECDC, 2024; Salam et al., 2023) (Table 4).

## 9 Conclusion

*K. pneumoniae* has emerged as one of the most formidable bacterial pathogens of the modern era, driven by the convergence of extensive antimicrobial resistance, high virulence potential, and remarkable genomic adaptability. Once primarily regarded as an opportunistic, hospital-associated organism, *K. pneumoniae* now poses a significant global health threat due to the rapid spread of MDR, XDR, and CRKP strains, as well as the parallel rise of hvKP. The pathogen's success is rooted in a complex network of virulence determinants, including capsular polysaccharides, siderophores, fimbriae, LPS, and diverse secretion systems, that collectively promote immune evasion, environmental persistence, biofilm formation, and efficient human colonization. The emergence of hybrid CR-hvKP strains that combine high resistance with hypervirulence further complicates disease management, leading to severe clinical outcomes, rapid dissemination, and limited therapeutic options.

AMR mechanisms in *K. pneumoniae* are multifactorial and increasingly sophisticated. These include intrinsic factors such as efflux pumps and reduced membrane permeability, as well as acquired mechanisms mediated by mobile genetic elements that enable rapid horizontal gene transfer. β-lactamase production remains a dominant driver of resistance, with ESBLs (e.g., TEM, SHV, CTX-M) and carbapenemases (e.g., KPC, NDM, OXA-48) contributing to global outbreaks and treatment failures. The spread of plasmid-borne *mcr* genes has further undermined the utility of colistin, one of the last-resort antibiotics, raising concerns about the emergence of pan-resistant strains. These developments have resulted in elevated morbidity, mortality, prolonged hospitalization, and mounting economic burdens on healthcare systems worldwide.

Clinically, the pathogen's impact is substantial across both healthcare-associated and community-acquired infections. Vulnerable populations, including ICU patients, immunocompromised individuals, neonates, and the elderly, face the highest risks, with severe infections such as liver abscesses, meningitis, pneumonia, and septicemia increasingly documented across the globe. Diagnostic limitations, particularly in detecting emerging resistance genes and hypervirulent markers, further hinder timely and appropriate treatment.

Addressing the expanding threat of *K. pneumoniae* requires a multipronged global strategy. Strengthening antimicrobial stewardship, improving diagnostic capacity, investing in novel therapeutics, including β-lactamase inhibitors, bacteriophages, monoclonal antibodies, and antivirulence agents, and implementing rigorous infection control measures are essential steps. Enhanced international surveillance and coordinated public health interventions will be critical to curbing the spread of high-risk clones and resistance determinants. Ultimately, the escalating challenge posed by *K. pneumoniae* underscores the urgency of sustained scientific, clinical, and policy-driven efforts to preserve the effectiveness of current treatments and prevent a further slide toward a post-antibiotic era.
